# Diagnostic Accuracy of Screening Tests for Diabetic Peripheral Neuropathy: An Umbrella Review

**DOI:** 10.1155/jdr/5902036

**Published:** 2024-12-04

**Authors:** María Mogilevskaya, Mariana Gaviria-Carrillo, John Edwin Feliciano-Alfonso, Ana M. Barragan, Carlos A. Calderon-Ospina, Mauricio O. Nava-Mesa

**Affiliations:** ^1^Neuroscience Research Group (NeURos), Neurovitae-UR Neuroscience Center, Institute of Translational Medicine (IMT), School of Medicine and Health Sciences, Universidad del Rosario, Bogotá 111221, Colombia; ^2^Internal Medicine Department, School of Medicine, Universidad Nacional de Colombia, Bogotá 111321, Colombia; ^3^Public Health Research Group, School of Medicine and Health Sciences, Universidad del Rosario, Bogotá, Colombia; ^4^School of Medicine and Health Sciences, Center for Research in Genetics and Genomics (CIGGUR), Institute of Translational Medicine (IMT), Universidad del Rosario, Bogotá, Distrito Capital, Colombia; ^5^Research Group in Applied Biomedical Sciences (UR Biomed), School of Medicine and Health Sciences, Universidad del Rosario, Bogotá 111221, Colombia

**Keywords:** diabetic neuropathy, diagnosis, early detection, peripheral neuropathy, screening tools

## Abstract

Peripheral neuropathy is a common cause of morbidity in diabetes. Despite recent advancements in early diagnosis methods, there is a need for practical, highly sensitive, and cost-effective screening methods in clinical practice. This study summarizes evidence from systematic reviews and meta-analyses on the diagnostic accuracy of validated screening methods for diabetic peripheral neuropathy. Two independent reviewers assessed methodological quality and bias using AMSTAR and ROBIS tools. Seven reviews with 19,531 participants were included. The monofilament test showed inconsistent sensitivity (*S*: 0.53–0.93) and specificity (Sp: 0.64–1.00), along with high variability in its application. Neuropad exhibited high *S* (86%, 95% CI 79–91). However, variations in the interpretation of results across the included studies may have impacted its Sp (65%, 95% CI 51–76). The Ipswich touch test exhibited adequate diagnostic accuracy (*S*: 0.77, Sp: 0.96, DOR: 75.24) but lacked comparison with gold standard tests. In vibration perception studies, the biothesiometer outperformed the tuning fork (*S*: 0.61–0.80 vs. 0.10–0.46). In general, heterogeneity was observed due to varied reference tests, thresholds, and patient differences. The development of automated analysis methods, as well as determination of predictive value of the combination of screening tools, is needed for further studies. Based on the study results, we suggest that clinicians should select screening tools tailored to their patient population, clinical setting, and available resources, as no single test can be universally recommended for all clinical scenarios.

## 1. Introduction

According to the World Health Organization (WHO), approximately 422 million people worldwide have diabetes [1] with a prevalence of 10.5% among the adult population [2]. Nowadays, 1.5 million global deaths are attributed to complications from diabetes [1]. Additionally, the number of diabetes cases is expected to rise, with an estimated 578 million cases by 2030 and 700 million by 2045 [1].

Diabetes leads to severe complications within a range of body's systems such as the kidney, cardiovascular system, and retina [3]. Damage to the nervous system is considered one of the most frequent chronic complications, with prevalence varying between 10% and 66% [4–6]. Furthermore, 50% of adults with diabetes will develop diabetic peripheral neuropathy (DPN) [1]. There are different forms of neuropathy, caused by diffuse and focal nervous system damage. The most common type of diabetic neuropathy is distal symmetric polyneuropathy (DSPN), which shows a “stocking and glove” pattern due to damage to the longest sensory axons. Damage then spreads to autonomic axons and finally to motor axons, while neuronal cell bodies remain relatively intact (reviewed in [7]). Other forms of neuropathies can occur such as small-fiber neuropathy, autonomic neuropathies, focal neuropathies, and polyradiculopathy [7]. Diabetic neuropathies have a negative impact on the quality of life and have been associated to cardiovascular morbidity, cardiac death, and nonfatal myocardial infarction [8]. Furthermore, the annual costs of diabetic neuropathy and its complications are high [7].

Despite their high rates of morbidity, mortality, and cost, diabetic neuropathies are often overlooked and underdiagnosed. This may be partly due to a lack of consensus on screening and diagnostic methods. Moreover, in some countries, simple diagnostic tools such as the tuning fork are only available in a specialist setting [8]. Currently, screening for diabetic neuropathy is highly recommended at diagnosis and annually for patients with Type 2 diabetes mellitus (T2DM) and starting 5 years after diagnosis for patients with Type 1 diabetes mellitus (T1DM) [7]. The screening tests should be rapid, reliable, and simple to obtain a broad population application. Different tests based on specific instruments or devices, such as physical examination, medical questions, and composite scoring systems, can be performed to detect diabetic neuropathy. For instance, the use of the 10-g monofilament (MF), vibration testing with a 128-Hz tuning fork, assessment of deep tendon reflexes, and the Michigan Neuropathy Screening Instrument (MNSI), among others, is widespread [7]. Although various narrative reviews describe and discuss in detail many of the early diagnosis methods [9, 10], as well as potential biomarkers [11], there is vast variability in sensitivity (*S*) and specificity (Sp) values, as well as variations in the expert consensus on screening tools and clinical diagnosis methods [8]. This umbrella review seeks to compile and evaluate evidence from various systematic reviews and meta-analyses of validated screening tests for DPN, focusing on their diagnostic accuracy.

## 2. Methods

The protocol for this umbrella review was registered in PROSPERO (CRD42023464186). We conducted this umbrella review in accordance with the Preferred Reporting Items for Systematic Reviews and Meta-Analyses (PRISMA) declaration criteria and the current recommendations of the Cochrane Collaboration (overview of reviews (Cochrane description: [12])). The reporting strategy for the present review followed the PRISMA guideline.

### 2.1. Review Question/Objective

This review is aimed at conducting a rigorous systematic search and comprehensively synthesizing evidence from existing systematic reviews and meta-analyses. The objective is to offer a reliable understanding of the diagnostic utility of screening tests for DPN compared to the established gold standard and other reference tests.

This review also encompasses the examination of supplementary outcomes, including positive predictive value (PPV), negative predictive value (NPV), positive likelihood ratio (PLR), negative likelihood ratio (NLR), diagnostic odds ratio (DOR), and receiver operating characteristic (ROC) analysis (area under the curve (AUC) comparison), for each of the screening tests.

### 2.2. Search Strategy

We conducted a thorough literature search using the following electronic databases: Ovid MEDLINE, Embase, Cochrane Database of Systematic Reviews, and LILACS. The initial search was performed on April 3, 2023, followed by subsequent searches on April 4 and April 6, 2023. No restrictions were imposed regarding publication dates or language (a detailed search strategy can be found in Appendix S1). Additionally, we conducted a manual search by scrutinizing the reference lists of the selected articles and utilizing the Google search engine.

### 2.3. Inclusion Criteria

#### 2.3.1. Types of Reviews

In this umbrella review, we have included systematic reviews and meta-analyses that encompassed information on the accuracy of screening tests for DPN.

#### 2.3.2. Participants

Studies on patients with either Type 1 or Type 2 diabetes were considered. Moreover, we included studies involving diabetic patients with either suspected or confirmed DPN. We also incorporated studies in patients with diabetes whose medical history presented nonpainful signs of DSPN and/or neuropathic pain symptoms characteristic of DSPN as well as studies encompassing diabetic patients who were asymptomatic for DPN. There were no restrictions concerning participants ethnicity or gender.

### 2.4. Exclusion Criteria

The exclusion criteria for this study were as follows:

● Studies involving patients with prediabetes.
● Studies that included patients with neuropathy originating from causes unrelated to diabetes.
● Studies lacking sufficient information on the Sp, *S*, and other outcomes of screening tests.
● Studies whose primary objectives did not involve evaluating the accuracy of screening tests for DPN and articles covering unrelated subjects (i.e., prevalence of the test).

### 2.5. Selection Process

During the study selection process, we began by eliminating any duplicate entries. Subsequently, two independent reviewers screened the studies identified in the literature search and assessed them based on their titles and abstracts. They addressed any discrepancies through discussion and, when necessary, engaged a third reviewer to achieve a consensus.

**Figure 1:**
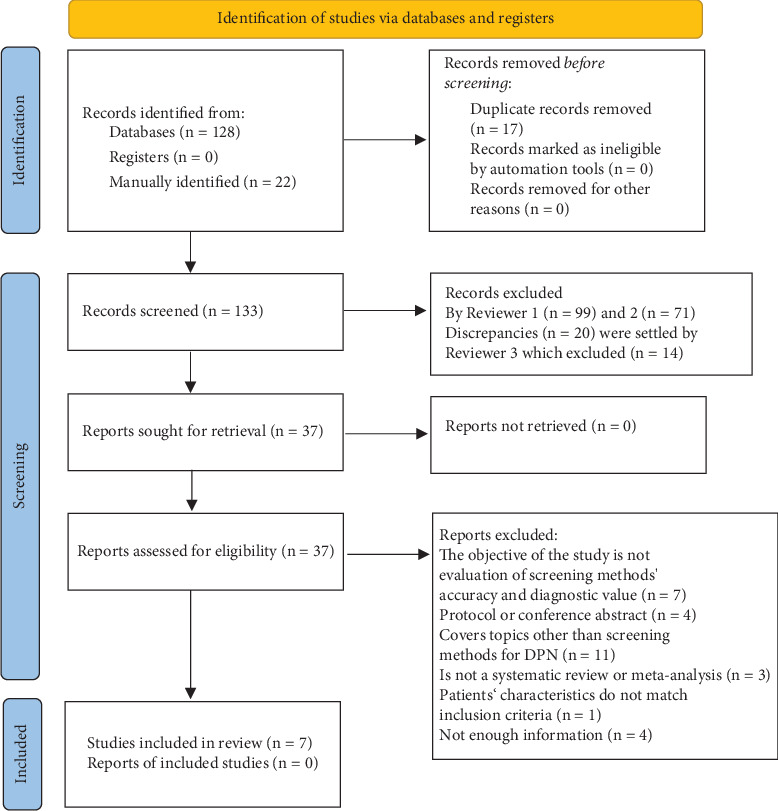
Flowchart of the selection process.

Following this initial screening, we retrieved the full texts of the selected articles. Again, two reviewers independently assessed these articles against the predetermined inclusion criteria. For transparency, we provided reasons for excluding each study in Figure 1. Any disagreements during this phase were resolved through discussion or, when required, with input from a third party.

### 2.6. Data Extraction

After the selection process was completed, the chosen articles underwent a meticulous data extraction process. Two reviewers independently extracted the specified variables from each of the selected articles. Finally, as a concluding step, a third reviewer verified the extracted data for both accuracy and completeness.

The following groups of variables were extracted whenever available:

● Study characteristics such as author(s), country, year, study design, sample size, risk of bias, quality report, and limitations reported by the authors.
● Participant characteristics including the percentage of female participants, age, diabetes duration, type of diabetes, and glycated hemoglobin (HbA1c) value.
● Characteristics of the screening test(s) such as definition of abnormal measurement, setting of the test (including time, place, and test performer), *S*, Sp reference test or gold standard comparison, PPV, NPV, false-negative rate, false-positive rate, diagnostic accuracy, and ROC curve data.

### 2.7. Assessment of Methodological Quality

The assessment of the risk of bias and quality reports was carried out by two independent evaluators utilizing the risk of bias in systematic reviews (ROBIS) [13] and assessment of multiple systematic reviews (AMSTAR 2.0) [14] tools for each article. Any discrepancies that arose during this assessment process were resolved through discussion, and if required, a third reviewer was consulted for resolution.

## 3. Results

### 3.1. Study Selection

The study selection process is illustrated in Figure 1. In addition to the initial database search, which identified 128 articles, we manually searched the reference lists of the included papers. This manual search, along with a query using the Google search engine, identified 22 additional papers. The list of excluded full-text articles with the reasons for exclusion is detailed in Supporting Information S1 list of excluded full texts. In total, seven studies were included in this umbrella review [15–21].

### 3.2. Main Characteristics of the Systematic Reviews and/or Meta-Analyses Included

The main characteristics of the included studies can be found in Table 1. Of the seven included studies, three performed meta-analyses [15, 16, 21] and four [17–20] comprised only systematic review. The number of studies included in the reviews varied between three [17] and 19 [21] primary studies. Though most of the studies did not specify the study designs of the included articles, Zhao et al. [15] state that only original reports were included, excluding case reports, conference papers, case series, and reviews. Furthermore, Tsapas et al. [16] indicated that they included both cohort-type and case–control-type accuracy studies and excluded prognostic accuracy studies.

### 3.3. Methodological Quality and Risk of Bias

**Figure 2:**
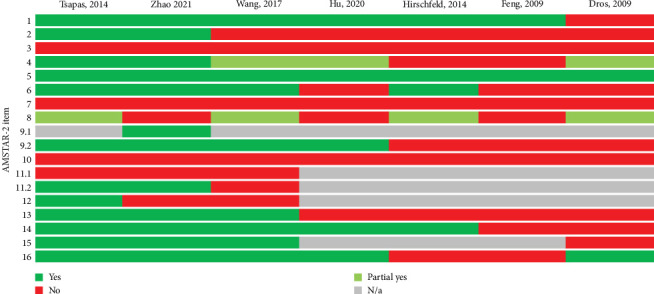
AMSTAR-2 tool assessment results by item.

The results obtained through application of the AMSTAR tool are depicted in Figure 2. Overall confidence in the results of the included reviews was low or critically low. Detailed breakdown of AMSTAR tool results can be found in Supporting Information AMSTAR.

Regarding the application of the ROBIS tool, it was found that the risk of bias in the systematic review was low in the case of the studies by Zhao et al. [15], Wang et al. [21], and Tsapas et al. [16]. The evaluators assessed the risk of bias as high in the study conducted by Feng, Schlösser, and Sumpio [18], citing concerns in Domains 2, 3, and 4. They also identified a high risk of bias in the study by Hu, Koh, and Teo [20], noting concerns across all domains. Finally, the risk of bias was unclear in the case of the studies by Hirschfeld et al. [19] and Dros et al. [17] due to lack of information in several domains (Table 2).

The details regarding the methodological quality assessment of included studies can be found in Table S1. In total, six of the included studies reported evaluating the risk of bias in the primary studies. One study [18] did not provide any risk-of-bias evaluation. Additionally, two studies [15, 17] did not utilize the current version of the QUADAS tool (QUADAS-2) but instead used the previous version.

### 3.4. Participants

Globally, the selected systematic reviews and meta-analysis included 19531 participants. This number of participants varied from 641 to 8365 between studies, with ranges of age from 12.9 to 64. Most of the studies included adults with Type 2 diabetes, few patients with Type 1 diabetes [16, 17, 20, 21], and only one of the young patients with Type 1 insulin-dependent diabetes mellitus [19]. However, in some reviews, age and type of diabetes were not reported.

The information about the origin of primary studies was occasionally provided and included countries such as Australia, Austria, Brazil, Canada, France, Greece, Germany, India, Iran, Italy, Korea, Portugal, Saudi Arabia, Serbia, Spain, Sweden, Turkey, United Kingdom, and United States. None of the studies selected include information from Africa.

Primary studies included in the reviews recruited participants from diabetes centers, clinics, and hospitals; few of them were from diabetes outpatient centers and population surveys. Severity was occasionally reported according to the presence of ulcers and duration of the disease or depending on some clinical scales (i.e., neuropathy disability score (NDS) and diabetic neuropathy index (DNI)); however, it is difficult to correlate results of the screening test with grade of nerve injury.

### 3.5. Methods of Analysis

In this umbrella review, four of the seven included reviews ([17–20]) presented data in both narrative and tabular formats. This approach was necessary due to the heterogeneity in testing procedures observed in the primary studies, statistical limitations, and other methodological differences among the studies. Three systematic reviews were meta-analyzed from primary studies ([15, 16, 21]) assessing the Ipswich touch test (IpTT), Neuropad test, and MF test, respectively. The methods of performing the screening and reference tests were similar ([15, 21]). In the study by Wang et al., the authors chose a threshold that was closest to the “half cutoff” threshold used in other studies. They included only research that used neuroconduction studies (NCSs) as the reference standard for data synthesis. In the same study, the authors employed a hierarchical summary receiver operating characteristic (HSROC) model to conduct a meta-analysis of diagnostic accuracy. This and additional analyses were presented in forest plots created using Meta-DiSc. Zhao et al. [15] performed a meta-analysis of screening accuracy based on the quality effect model, and they evaluated the relationship between *S* and Sp using summary receiver operating characteristic (SROC) analysis. The same authors considered an index of inconsistency (*I*^2^) ≥ 50% as significant heterogeneity. Meanwhile, in the study of Wang et al. [21], statistical tests such as chi-square, Cochran-*Q*, and *I*^2^ were used to quantify the amount of heterogeneity. In the study by Tsapas et al. [16], the authors used HSROC models implemented through user-written modules (metandi, midas) to assess diagnostic accuracy and heterogeneity. They evaluated clinical utility using Fagan's nomograms based on Bayes' theorem and employed meta-regression analyses to investigate the influence of the reference standard used and prevalence as potential moderators of diagnostic accuracy.

### 3.6. Index Tests

The index tests are summarized in Table 3. Four articles discuss the utilization of MF testing for the detection of DPN ([17–19, 21]). Wang et al. [21] provide data regarding the testing site, revealing that the most common location for testing is the great toe. Additionally, they offer insights into the type of MF used for screening and report findings from 19 studies. Hirschfeld et al. [19] also include information about testing sites; however, they include only two studies that evaluated *S* to light touch. Feng, Schlösser, and Sumpio [18] highlight that the most sensitive method involves testing the third and fifth metatarsal heads on each foot upon assessing 16 studies.

In addition to MF testing, Hirschfeld et al. [19] provide data on the biothesiometer and tuning fork, encompassing findings from five studies employing these methods. Two other studies ([15, 20]) report on the IpTT as a screening tool for DPN. One of the included studies, Tsapas et al. [16], describes a color-changing adhesive indicator test applied to the sole of the foot to identify sweating called Neuropad.

### 3.7. Reference Tests

Table 3 describes reference tests used in the included studies. There was some heterogeneity regarding reference tests employed in the studies. Four studies included reports that used nerve conduction study as a reference standard ([17–19, 21]). Some of the reviews reported a combination of reference tests such as vibration perception threshold (VPT), NDS, MNSI, San Antonio consensus evaluation, detailed neurological assessment (DNA), DNI, Hoffmann's reflex test (HRT), Michigan diabetic neuropathy score (MDNS), 10-g MF, and combination of signs and symptoms [16, 18, 20, 21]. One of the studies reported 10-g MF as a sole reference standard [15].

### 3.8. Findings

The findings from the included systematic reviews, search sources, and data frame are summarized narratively and presented through tables and graphics. The detailed information includes the following:

• The type of study or review,
• Characteristics of the primary studies (such as population size, sex, and age distribution),
• Features of diabetes (including type and duration), and
• Aspects of neuropathy.

Additionally, it outlines the setting and specific characteristics of each screening and diagnostic test. *S*, Sp, PLR, NLR, and DOR were evaluated, and additional data were calculated when sufficient information was available (false negative, false positive, true negative, and true positive). Finally, the area under the ROC (AUROC) curve was extracted (see Table 4).

### 3.9. Outcomes

The findings were categorized by subgroups based on the type of test employed: MF, vibration threshold (biothesiometer and tuning fork), IpTT, or sweat indicator (Neuropad), and by the type of comparator. Summary or pooled measures were disclosed if data was available in the systematic reviews; otherwise, the minimum (min) and maximum (max) values reported in each systematic review were presented.

#### 3.9.1. MF Compared to NCS

Information regarding the comparison of MF methods to NCSs was extracted from five systematic reviews. Only one of these reviews conducted a meta-analysis [21], revealing a *S* of 0.53 (95% confidence interval (CI) 0.32–0.74, *I*^2^ 96.4%). In the remaining reviews, median (Me), min, and max values from the included studies are provided as follows: Feng, Schlösser, and Sumpio [18] reported a Me of 0.57 (range: min 0.44; max 0.68) to 0.93 (range: min 0.77; max 0.99 from two studies; 1065 patients), Hirschfeld et al. [19] ranged from 0.19 (min 0.10; max 0.33) to 0.73 (min 0.46; max 0.89), and Dros et al. [17] ranged from 0.41 (min 0.36; max 0.46) to 0.93 (min 0.77; max 0.99).

Reported Sp in the studies was as follows: Wang et al. [21] documented a Sp of 0.88 (95% CI 0.78–0.94) in their meta-analysis. Hirschfeld et al. [19] provided values of 0.64 (range: min 0.46 to max 0.78) and 0.87 (range: min 0.70 to max 0.95), while Dros et al. [17] reported a spectrum from 0.95 (range: min 0.86 to max 0.99) to 1.00 (range: min 0.63 to max 1).

The effectiveness of the MF test was expressed by the DOR, which indicates the odds of the test being positive for someone with the disease compared to the odds of the test being positive for someone without it. A higher DOR value corresponds to a higher AUC. However, the reports showed variation in these results. Wang et al. [21] reported 8.62 (95% CI 4.69–15.84), while in the study by Hirschfeld et al. [19], the values ranged from 0.43 (min 0.15 to max 1.24) to 19.00 (min 3.61 to max 99.94).

#### 3.9.2. MF Compared to Other Reference Tests

The 5.07-/10-g Semmes Weinstein monofilament examination (SWME) served as the index test in the systematic review conducted by Feng, Schlösser, and Sumpio in 2009 [18] and was compared with five additional reference standards. *S* and Sp were reported in all studies and compared in DNA: *S*: 0.30 from one study, involving 82 patients; history of ulceration: *S*: 0.51–0.95 from four studies, encompassing 2532 patients; Sp: 0.65–0.85; HRT: *S*: 1 from one study, including 340 patients; San Antonio consensus evaluation: *S*: 0.42 from one study, with 305 patients; and vibration threshold with biothesiometer: *S*: 0.47 from one study, involving 250 patients.

#### 3.9.3. VPT Tests Compared to NCS

Hirschfeld et al.'s 2014 systematic review [19] provided insights into VPT techniques. The tests included the biothesiometer, with sensitivity (*S*) ranging from 0.61 (from 0.46 to 0.74) to 0.80 (from 0.60 to 0.91) from two studies, Sp ranging from 0.64 (from 0.46 to 0.78) to 0.76 (from 0.51 to 0.91), and DOR ranging from 2.86 (from 1.10 to 7.39) to 13.5 (2.78 to 65.5). Additionally, the tuning fork exhibited sensibility ranging from 0.01 (from 0.00 to 0.10) to 0.19 (from 0.06 to 0.46) from three studies, Sp ranging from 0.87 (from 0.70 to 0.95) to 0.99 (from 0.87 to 0.99), and a DOR of 0.74 (from 0.01 to 38.65) to 2.29 (from 0.04 to 120.75). All tests were compared to NCSs.

#### 3.9.4. IpTT

Two systematic reviews were identified, evaluating light touch test in comparison with MF [15, 20], NDS ≥ 6, and VPT [20]. The study by Zhao et al. [15] conducted a meta-analysis of seven studies. The findings indicated that the IpTT cannot effectively rule out DPN. However, it can confirm the condition with a combined *S* of 0.77 (95% CI 0.69–0.84, *I*^2^ = 40.5%) and a Sp of 0.96 (95% CI 0.93–0.98). The DOR was also reported as 75.24 (95% CI 39.90–141.89).

Concerning Hu, Koh, and Teo's 2021 systematic review [20], in comparison with a 10-g MF, the reported *S* ranged from 0.51 to 0.83 (three studies; 932 patients), and Sp ranged from 0.96 to 0.98. When compared with NDS, the *S* ranged from 0.53 to 1 (two studies; 434 patients), and Sp ranged from 0.90 to 0.97. In comparison with VPT (≥ 25 V), the *S* ranged from 0.76 to 1 (three studies; 699 patients), and Sp ranged from 0.92 to 0.97.

#### 3.9.5. Plantar Sweat Indicator—Neuropad

This technique was evaluated only in one systematic review and meta-analysis [16]. In this study, it was compared against four distinct reference standards: a combination of signs and symptoms ± conduction studies, DNI (> 2 or unclear), MDNS (> 0), and NDS (≥ 3 or 5 or 6). Following the HSROC analysis encompassing all 18 studies, the authors report the summary *S* of 86% (range 43–100, 95% CI 79–91) and Sp of 65% (range 22–100, 95% CI 51–76).

A summary of the main data and outcomes derived from the reviews is presented in Table 4.

## 4. Discussion

In the current umbrella review, we assessed the accuracy of five screening tests by analyzing seven published systematic reviews and meta-analyses. For each test under consideration, we evaluated 10 outcomes, encompassing *S*, Sp, positive and NPVs, DOR, and positive and NLRs. When data were accessible, false-negative and false-positive rates for the screening tests under analysis were computed. Upon evaluating methodological features, it was determined that the three meta-analyses meet several quality criteria. However, none of them fulfills all 16 AMSTAR-2 criteria. While the AMSTAR-2 tool has not been validated for evaluating the quality of systematic reviews on diagnostic test accuracy (DAT), the determination of overall study quality relied on the presence or absence of critical domains [14]. The overall confidence in the results of the included reviews was low or critically low. The implementation of AMSTAR in this review adhered to traditional guidelines, despite acknowledging certain limitations [22]. In the analysis with the ROBIS tool, it was considered that the meta-analysis of Tsapas et al. [16] and Wang et al. [21] has low risk of bias; meanwhile, other systematic reviews and meta-analysis have intermediate level. While the methodological recommendations for conducting integrative studies on diagnostic tests are still under development [23], we reported the quality confidence and risk of bias assessment as a pragmatic approach to enhance the transparency and quality of the review. When assessed against various reference standards, the validity measures, specifically *S* and Sp, for MF techniques generally indicate higher Sp than *S*. However, this trend is inverted in the case of the HRT, where *S* exceeds Sp. As for the measure of effectiveness, the DOR [24], studies reveal considerable discrepancies. As for vibration threshold tests, no consistent pattern was identified; both *S* and DOR exhibited complete divergence. Regarding IpTTs, the observed trend indicated *S*s greater than 70% and Sps greater than 90%, coupled with a highly suggestive DOR of the disease when a positive result was obtained.

Research involving the IpTT has not compared it to established gold standard assessments, such as NCSs or biopsies; instead, it has been compared against other screening tests. The studies by Zhao et al. [15] and Hu, Koh, and Teo [20], which use the MF as a reference test, provide a rationale for comparing IpTT with a validated screening tool that is simple, quick, and noninvasive, preserving the critical characteristics of an effective screening method. This approach is presented by authors as a practical and accessible means of detecting DPN, especially in settings with limited access to advanced diagnostic tools. Additionally, the meta-analysis indicates that IpTT exhibits acceptable *S* and even higher Sp when compared to MF. Therefore, we conclude that comparing multiple index tests with various reference tests indicates that the understanding of screening for peripheral neuropathy is still in an exploratory phase [25]. Studies of this nature are often aimed at evaluating the feasibility and performance of new diagnostic tools by administering several tests to the same participants. This approach allows researchers to observe the tests' performance under different conditions and generate hypotheses. Regarding the interpretation of results and their implications for clinical practice, it is advisable to use multiple diagnostic tests in a complementary manner, depending on available resources. Applying these tests in specific contexts, along with continuous monitoring and reevaluation, helps to reduce uncertainty.

In our umbrella review, multiple studies on traditional screening methods highlight various challenges associated with their utilization in screening for DPN. The MF test, in particular, exhibits significant variability in methodologies. Despite the widespread use and endorsement of MF tests in various clinical guidelines, there remains a lack of consensus on the optimal location, number of sites [26], and threshold values for DPN diagnosis [18, 21]. Moreover, commercially available MF kits might differ in their performance [27].

Dros et al. [17] note that the wide ranges in Sp and *S* reported in different studies may stem from variations in how the MF test is applied including the number of tests conducted and the sites used. Additionally, differences in the interpretation of the test thresholds and the characteristics of the study populations may also contribute to these variations. These nuances in methodology underscore the importance of standardization and consistency in administering and interpreting the MF test for more reliable and comparable results across studies.

Hirschfeld et al.'s study in the pediatric population [19] shows low diagnostic utility for traditional methods (tuning fork and 10-g MF). However, biothesiometry and finer (1 g) MFs exhibit acceptable diagnostic utilities. Despite potential bias of this review towards overly negative results, the conclusions on the limited utility of noninvasive screening methods persist, even after excluding certain imputed studies.

We have included three studies that investigate newer screening methods for DPN. The IpTT is an inpatient screening method that does not require specialized tools or extensive training and has versatile applicability across time, location, and operator expertise. However, concerns about its diagnostic utility have been raised due to methodological variations and reference standard issues in previous research [28].

In a 2021 review by Hu, Koh, and Teo [20], the IpTT is acknowledged for its notable Sp. Nevertheless, the review emphasizes the need for a more robust research base to support its clinical application. The limited evidence may explain why the IpTT is exclusively recommended in the International Working Group on the Diabetic Foot (IWGDF) guidelines for diabetic foot screenings when the 10-g MF is unavailable [29]. Despite recognizing the IpTT's high Sp, Zhao et al.'s 2021 review [15] stresses the urgency of strengthening the existing research foundation to enhance credibility and broaden recommended applications.

Neuropad, a sweat production measuring indicator plaster [30], lacks standalone diagnostic accuracy for confirming diabetic neuropathy, as emphasized in Tsapas et al.'s study [16]. Despite that, it proves valuable in clinical setting triage, aiding in the identification of individuals who need further assessment by a specialist team.

Given that anhidrosis can occur in patients with peripheral neuropathy, several additional screening methods beyond Neuropad are available. These include Sudoscan and the quantitative sudomotor axon reflex test (QSART), designed to detect this form of autonomic dysfunction. Those methods have the advantage of assessing small autonomic C-fiber function in an early manner that is difficult to detect with other tests, such as the IpTT or NCSs (which detect neuropathy in large fibers) [9]. Additional advantages are the easy application, noninvasibility, and availability in outpatient settings [10]. QSART has some problems that need to be considered as a fully validated screening method because of higher technical requirements and measurement variability, making it uncomfortable and time-consuming [31]. Neuropad and Sudoscan have some limitations because of variations in cutoff values and variations in the detection time and anatomic location applied. In the case of Sudoscan, there are reported intersubject variability and varied interpretations of the results that may affect their Sp [9, 32].

While our search did not yield any systematic reviews or meta-analyses detailing the use of composing scoring tests (such as questionnaires with symptoms and signs) for diabetic neuropathy screening, these tests have demonstrated reasonably high *S* and Sp. In a 2015 study by Xiong et al. [33], the neuropathy symptom and change (NSC) score exhibited a *S* of 85.96% but a lower Sp of 77.03%. The neuropathy impairment score (NIS) showed a relatively low *S* of 59.65% but higher Sp at 98.65%. Similarly, the MNSI demonstrated *S* of 70.18% and Sp of 98.65%, all in comparison with nerve conduction velocity (NCV).

Because they are not fully validated screening methods for neuropathy, we have excluded two studies on nerve elastography for diagnosing and detecting DPN. This technique assesses wave propagation through specific tissue, with wave speed directly correlating to tissue stiffness. In individuals with DPN, affected nerve stiffness is reported to be higher compared to control subjects [34]. In a recent meta-analysis by Chen et al. in 2022 [35], supersonic shear wave imaging (SSI) was highlighted, exhibiting a summary *S* of 80% (95% CI: 73%–86%) and a Sp of 86% (95% CI: 82%–89%) for diagnosing DPN through tibial nerve stiffness imaging. However, no cutoff values were established, other nerves commonly affected in DPN were not considered, and patient characteristics such as height and age were noted to potentially influence nerve stiffness. Consequently, the authors conclude that a prospective, multicenter investigation with a large sample size is warranted.

In a 2022 study by Dong et al. [34], shear wave elastography (SWE) demonstrated a summary *S* and Sp for tibial nerve stiffness of 75% (95% CI: 68%–80%) and 86% (95% CI: 80%–90%), respectively. The summary AUROC was 0.84 (95% CI: 0.81–0.87) for diagnosing DPN. While the *S* and Sp of elastography may be comparable to the MF technique in our study, the promising results notwithstanding both SSI and SWE do not meet the definition and criteria of a screening test for DPN: easy to perform and interpret [36].

Although our search strategy identified several studies [37, 38] on corneal confocal microscopy (CCM), none met the inclusion criteria. CCM is a technique used to define small A*δ*- and C-fiber structure and damage [39]. It is now evident that CCM holds promise as a tool for detecting both subclinical and clinical DPN [38]. Moreover, it can predict individuals at risk of developing DPN [37]. A 2022 study by Gad et al. [38] demonstrated a significant reduction in corneal nerve fiber density (CNFD), corneal nerve branch density (CNBD), inferior whorl length (IWL), and corneal nerve fiber length (CNFL) in individuals with DPN. Another study reported that setting thresholds for CNFL at upper (> 15.8) and lower (< 11.8) values yields 91% *S* and 93% Sp for diagnosing DPN in individuals with Type 1 diabetes [40]. A 2022 systematic review by Galosi et al. [37] provides a narrative overview of CCM use for DSPN detection. The authors point out that, despite some available evidence on CNFL assessment, diagnostic thresholds for other CCM measures have not yet been established.

In this umbrella review, we have observed that heterogeneity stems from several factors, including differences in the reference tests, patient populations, and the thresholds applied in the studies analyzed. These variations can influence the reliability and applicability of certain tests in specific clinical settings or patient groups. While some screening tools, such as the IpTT, demonstrated high Sp (96%) and moderate *S* (77%), their utility may be more appropriate in inpatient or primary care settings where quick, resource-light assessments are needed. On the other hand, tools like the biothesiometer, with consistently higher diagnostic accuracy (*S* ranging from 61% to 80%), may be better suited for specialized care or when there is access to more advanced equipment. This suggests that some tests might be more reliable in settings where the prevalence of neuropathy is higher or where more detailed diagnostic assessments are possible.

The effectiveness of screening methods for DPN is likely influenced by factors such as healthcare infrastructure, availability of diagnostic tools, and patient demographics, all of which vary significantly between regions. In regions with limited healthcare infrastructure, tools like the IpTT, which do not require specialized equipment, could be particularly valuable due to their simplicity and low cost. However, while IpTT has demonstrated high Sp, its *S* may not be sufficient for detecting early-stage neuropathy, particularly in populations with limited access to follow-up care. In contrast, more advanced tools like the biothesiometer or NCSs, though highly accurate, may be less feasible in low-resource settings due to their cost and the need for specialized equipment and trained personnel. Patient demographics, such as age distribution, prevalence of comorbidities, and genetic factors, might also play a role in the variability of test performance. For instance, older populations with longer disease duration or higher rates of comorbid conditions may present with more advanced neuropathy, which could influence the *S* of screening tools. Additionally, cultural factors, such as differences in health-seeking behaviors and accessibility to healthcare, may impact the timing of diagnosis and, consequently, the performance of early detection methods.

The combination of symptoms and clinical signs may increase the diagnostic accuracy of diabetic neuropathy. Different composite scoring systems, such as the MNSI and the Toronto Clinical Neuropathy Score (TCNS), have been widely used [9]. A recent study demonstrated that Sudoscan, combined with MNSI, could detect diabetic neuropathy with greater discrimination power than isolated tests or in combination with MF [41]. Additionally, another study found that the combination of the diabetic neuropathy symptom (DNS) questionnaire and ankle reflex testing provides higher diagnostic value for large-scale population screening in a cost-effective manner [42]. Emerging technologies based on artificial intelligence (AI) and automated data interfaces have the potential to further improve diagnostic accuracy for central and peripheral nervous system disorders by using large clinical and paraclinical databases in combination with specific disease biomarkers [43, 44]. For example, machine learning–based methodology has been used to enhance the diagnostic accuracy of carpal tunnel syndrome by considering individual, clinical, and sonographic characteristics [45]. At the same time, deep learning models have been developed for predicting chemotherapy-induced peripheral neuropathy [46]. These technologies may be applied to both validated and novel screening methods for diabetic neuropathy in the future.

## 5. Conclusions

It is necessary to develop different early detection tools to reduce the progression of DPN. Although early diagnosis methods have been postulated in recent years, as well as the development of different biomarkers, in everyday clinical practice, better screening methods that are easy to apply, highly sensitive, and cost-effective must be developed and implemented. In the present umbrella review, we focus on the diagnostic accuracy of methods previously validated as screening tests. According to the AMSTAR 2 tool, methodological quality was better in the studies concerning Neuropad and IpTT studies. Meanwhile, the studies with Neuropad and MF 10 g have a low risk of bias.

MF has the advantage of being highly available and easy to apply. However, it presents relatively low levels of *S* and Sp, as well as high variability and subjectivity. The study with Neuropad has adequate methodological quality and relatively good *S*; however, they may have variations in the interpretation of their results that may affect their Sp. IpTT has good diagnostic accuracy. However, the studies were not carried out comparing it with a gold standard test such as neuroconduction or biopsy; instead, they were compared with other screening tests. In vibration perception studies, better diagnostic accuracy levels are shown through the biothesiometer. Although CCM and elastography were not included in the data synthesis of the present umbrella review because they are not considered fully validated screening tests, they have high potential, considering these are rapid and noninvasive tools with higher levels of sensibility.

All tests evaluated in this study can be conducted in both outpatient and inpatient and administered by primary care physicians trained in the relevant techniques. While most tests require specific instruments, the IpTT is an exception, as it does not necessitate specialized equipment. In summary, future research should focus on assessing the performance of these diagnostic tools in various healthcare settings and across diverse patient populations. Tailoring screening strategies to each region's specific needs and resources could enhance the early detection and management of DPN on a global scale. Future clinical trials should provide insights into the clinical utility of different screening methods across diverse patient populations and settings.

The development of automated analysis methods—such as software and interfaces based on AI—is essential, as is determining the optimal combination of screening tools and biomarkers to enhance their predictive value. Progress in those analytics and their widespread distribution in outpatient settings will be required in further studies.

## Figures and Tables

**Table 1:** Main characteristics of included studies. Abbreviations: HbA1c, glycated hemoglobin; SR, systematic review; MA, meta-analysis.

**Study reference**	**Type of study**	**Number of included studies**	**Number of patients included**	**Age of participants (mean and range)**	**Percentage of female participants**	**Type of diabetes (T1D/T2D)**	**Diabetes duration (years)**	**HbA1c**	**Source of article funding, conflict of interest**
Zhao et al., [15]	SR and MA	7 SRs 5 MAs	SR 1510 MA 1162	NR	NR	NR	NR	NR	Funding: Natural Science Foundation of Hunan Province (Grant No. 2019JJ80087) Competing interests: None declared
Wang et al., [21]	SR and MA	19 SRs 8 MAs	SR 3566 MA 1377 (range: 37–478)	Range of mean: From 41.4 to 65	Ranging from 32.3% to 68.7%	T1D and T2D	Ranging from 6.1 to 38.3 years	NR	Funding: NR Conflicts of interest: None
Feng, Schlösser, and Sumpio, [18]	SR	30 used for qualitative analysis; 16 used for quantitative analysis	Qualitative analysis: 8365 Quantitative analysis: 5019	NR	NR	NR	NR	NR	Competing interests: None
Hirschfeld et al., [19]	SR	5	699	Range of mean: 12.9–15.4 years	Range: 38.6%–54.3%	T1D	Range of mean: 2.3–8.2	Range of mean: 6.3–10.7	Funding: German Research Foundation grant (HI 1472/3-1) Conflict of interest: None
Hu, [20]	SR	5	1280	Range of mean: 47.9–64.5	ND	T1D and T2D	Range of mean: 7.4–14.4	NR	Funding: No funding source Conflict of interest: None
Tsapas et al., [16]	SR and MA	18	3470	NR	NR	T1D (15%) and T2D (85%)	Ranging from newly diagnosed to 18 years of duration	Range of mean: 6.3%–8.3% (reported in a subset of studies)	Funding: No funding source Conflicts of interest: NT reports honoraria fees from Miro Verbandstoffe, Wiehl-Drabenderhöhe, Germany, outside the submitted work. The remaining authors declare no conflict of interest
Dros et al., [17]	SR	3	641	Range of mean: 54–59 years	Ranging from 34% to 54%	T2D and NR	NR	NR	Funding: The Netherlands Organisation for Health Research and Development (ZonMw) (4200.0018) Conflict of interest: NR

**Table 2:** Quality assessment of included studies using the ROBIS tool. Low: low risk of bias, high: high risk of bias, and unclear: unclear risk of bias.

**Reference**	**Study eligibility criteria**	**Identification and selection of synthesis**	**Data collection and study appraisal**	**Synthesis and findings**	**Risk of bias in the review**
Zhao et al., [21]	Low	Low	High	High	Low
Hirschfeld et al., [19]	Low	High	Low	High	Unclear
Wang et al., [21]	Low	Low	Low	Low	Low
Feng, Schlösser, and Sumpio, [18]	Low	High	High	High	High
Dros et al., [17]	High	Low	Low	Unclear	Unclear
Tsapas et al., [16]	Low	Low	Low	Low	Low
Hu, [20]	High	High	High	High	High

**Table 3:** Index and reference tests in included studies.

**Author, year**	**Index test(s) (screening)**	**Reference test**	**Primary studies included**	**SR**	**MA**	**(Sample size) Number of patients included**
Zhao et al., 2021 [15]	Ipswich touch test (IpTT)	MF	7 SRs 5 MAs	1	1	SR 1520 MA 1162
Wang et al., 2017 [21]	10-g MF	NCS, VPT, NDS, MNSI, numbness in the toes, and loss of ankle jerk	8 MAs	1	1	MA 1377 (range 37–478)
Feng, Schlösser, and Sumpio, 2009 [18]	5.07-/10-g Semmes Weinstein monofilament examination (SWME)	History of ulceration, NCS, San Antonio consensus evaluation, vibration threshold with biothesiometer, detailed neurological assessment, HRT	16	1	0	4574
Hirschfeld et al., 2014 [19]	Tuning fork (3 studies) Monofilament (2 studies) Biothesiometer (2 studies)	NCS	5	1	0	Range: 21–232
Hu, Koh, and Teo, 2021 [20]	Ipswich touch test (IpTT)	MF, VPT, NDS ≥ 6	5	1	1	1280
Tsapas et al., 2014 [16]	Neuropad (simple paster)	NDS, MDNS, combination of signs and score, combination of signs and symptoms, and conduction studies	18	1	1	3470
Dros et al., 2009 [17]	Monofilament testing with the 5.07-/10-g monofilament	NCS	3	1	0	641

Abbreviations: DNI, diabetic neuropathy index; HRT, Hoffmann's reflex test; MA, meta-analysis; MDNS, Michigan Diabetic Neuropathy Score; MF, monofilament; MNSI, Michigan Neuropathy Screening Instrument; NCS, nerve conduction studies; NDS, neuropathy disability score; SR, systematic review; VPT, vibration perception threshold.

**(a):** 

**Article reference**	**Screening test(s)**	**Sensitivity (95% CI only with MA)**	**Specificity (95% CI)**	**ROC curve (AUC)**	**PLR (95% CI)**	**NLR (95% CI)**
Zhao et al., [15]	Ipswich touch test (IpTT)	0.77 (0.69–0.84)	0.96 (0.93–0.98)	AUC = 0.897 (0.86–0.93)	18.06 (10.75–30.36)	0.24 (0.17–0.35)

Wang et al., [21]	10-g MF	0.53 (0.32–0.74) HSROC model	0.88 (0.78–0.94) HSROC model	AUC = 0.8158	4.56 (2.93–7.10) HSROC model	0.53 (0.35–0.81) HSROC model

Feng, Schlösser, and Sumpio, [18]	5.07-/10-g Semmes Weinstein monofilament examination (SWME)	SWME vs. reference test: 1. Nerve conduction study: Range: 57% (95% CI 44%–68%) to 93% (95% CI 77%–99% 2. History of ulceration: Range 51%–95% 3. San Antonio consensus evaluation: 42% 4. Vibration threshold with biothesiometer: 47% 5. Detailed neurological assessment: 30% 6. Hoffmann's reflex test: 100%	SWME vs. reference test: 1. Nerve conduction study: Range: 7% (95% CI: 64%–84%) to 100% (95% CI: 63%–100%) 2. History of ulceration: Range 65%–85% 3. San Antonio consensus evaluation: 98% 4. Vibration threshold with biothesiometer: 97% 5. Detailed neurological assessment: 93% 6. Hoffmann's reflex test: 87%	NR	NR	NR

Hirschfeld et al., [19]	Tuning fork (3 studies) Monofilament (2 studies) Biothesiometer (2 studies)	Tuning fork (3 studies): Range 0.01 (0.00–0.10) to 0.19 (0.06–0.46) Monofilament (2 studies): Range 0.19 (0.10–0.33) to 0.73 (0.46–0.89) Biothesiometer (2 studies): Range 0.61 (0.46–0.74) to 0.80 (0.60–0.91)	Tuning fork (3 studies): Range 0.87 (0.70–0.95) to 0.99 (0.87–0.99) Monofilament (2 studies): Range 0.64 (0.46–0.78) to 0.87 (0.70–0.95) Biothesiometer (2 studies): Range 0.64 (0.46–0.78) to 0.76 (0.51–0.91)	NA	Tuning fork (3 studies): Range 0.75 (0.01–36.83) to 2.25 (0.04–108.45) Monofilament (2 studies): Range 0.55 (0.25–1.17) to 5.84 (2.07–16.44) Biothesiometer (2 studies): Range 1.71 (1.02–2.88) to 3.44 (1.34–8.81)	Tuning fork (3 studies): Range 0.92 (0.68–1.24) to 1.00 (0.95–1.05) Monofilament (2 studies): Range 0.55 (0.25–1.17) to 5.84 (2.07–16.44) Biothesiometer (2 studies): Range 0.25 (0.10–0.61) to 0.59 (0.37–0.94)

Hu, [20]	Ipswich touch test (IpTT)	Sens. of IpTT vs. reference test: 1. 10-g monofilament: Range 51%–83.3% (3 studies; 932 patients) 2. Vibration perception threshold (VPT ≥ 25 V): 76%–100% (3 studies; 699 patients) 3. Neuropathy disability score (> 6): 53%–100% (2 studies; 434 studies)	Spec. of IpTT vs. reference test: 1. 10-g monofilament: Range 96.4%–98% (3 studies; 932 patients) 2. Vibration perception threshold (VPT ≥ 25 V): 92%–96.6% (3 studies; 699 patients) 3. Neuropathy disability score (> 6): 90.3%–97% (2 studies; 434 studies)	NR	PLR of IpTT vs. reference test: 1. 10-g monofilament: Range 0.49–30.19 (3 studies; 932 patients) 2. Vibration perception threshold (VPT ≥ 25 V): 10.58–77 (3 studies; 699 patients) 3. Neuropathy disability score (> 6): 10.3–17.67 (2 studies; 434 studies)	NLR of IpTT vs. reference test: 1. 10-g monofilament: Range 0.05–0.50 (3 studies; 932 patients) 2. Vibration perception threshold (VPT ≥ 25 V): 0–0.27 (3 studies; 699 patients) 3. Neuropathy disability score (> 6): 0–0.49 (2 studies; 434 studies)

Tsapas et al., [16]	Neuropad (simple plaster)	86% (range 43–100, 95% CI 79–91) vs. NDS (highest available threshold ≥ 6) 89% (range 43–100, 95% CI 78–95) (3 studies) vs. DNI 76%–94% (five studies) vs. MDNS 73%–97% (2 studies) vs. combination of signs and symptoms 69%–90% (2 studies)	65% (range 22–100, 95% CI 51–76) vs. NDS (highest available threshold ≥ 6) 59% (range 23–80, 95% CI 48–70) (3 studies) vs. DNI 22%–92% (five studies) vs. MDNS 90%–100% (2 studies) vs. combination of signs and symptoms 47%–65% (2 studies)	HSROC plot is reported, but area under the curve (AUC) was not calculated	2.44 (95% CI 1.68–3.55)	0.22 (95% CI 0.14–0.34)

Dros et al., [17]	Monofilament testing with the 5.07-/10-g monofilament	Range 40.9 (36–46) to 93.1 (77–99)	Range 94.9 (86–99) to 100 (63–100)	NR	Range 10. 6 (4.0–28.0) to 16.5 (1.1–245.0)	Range 0.07 (0.02–0.26) to 0.61 (0.56–0.67)

Abbreviations: AUC, area under the curve; NLR, negative likelihood ratio; PLR, positive likelihood ratio; ROC curve, receiver operating characteristic curve.

**(b):** 

**Article reference**	**DOR (95% CI)**	**Threshold**	**PPV**	**NPV**	**False-negative rate (**1 − **S)**	**False-positive rate (**1 − **Sp)**	**I** ^2^
Zhao et al., [15]	75.24 (39.90–141.89)	NR	NR	NR	0.23 (0.16–0.31)	0.04 (0.02–0.07)	40.5

Wang et al., [21]	8.62 (4.69–15.84)	Multiple thresholds were set in two studies. They selected only the threshold that was closest to the threshold value (“half cutoff” threshold) used in other studies for the meta-analysis; therefore, a total of 12 groups of data were part of the meta-analysis	NR	NR	0.47 (0.26–0.68) HSROC model	0.12 (0.06–0.22) HSROC model	*S* = 96.4% Sp = 93.7% PLR = 72.4% NLR = 94.4% DOR = 60.3%

Feng, Schlösser, and Sumpio, [18]	NR	• There was no consensus for this threshold • In the 16 selected studies, the diagnostic threshold was set as one incorrect answer or inability to detect one site	PPV of SWME vs. reference test: 1. Nerve conduction study: Range: 84% (95% CI 74%–90%) to 100% (95% CI 87%–100%) 2. History of ulceration: Range 16%–66% 3. San Antonio consensus evaluation: 86% 4. Vibration threshold with biothesiometer: 87% 5. Detailed neurological assessment: 80% 6. Hoffmann's reflex test: 68% patients	NPV of SWME vs. reference test: 1. Nerve conduction study: Range: 36% (95% CI: 29%–43%) to 94% (95% CI: 91%–96%) 2. History of ulceration: Range 73%–99% 3. San Antonio consensus evaluation: 84% 4. Vibration threshold with biothesiometer: 83% 5. Detailed neurological assessment: 58% 6. Hoffmann's reflex test: 100%	SWME vs. reference test: 1. Nerve conduction study: Range: 7% (95% CI 1%–23%) to 43% (95% CI 32%–56%) 2. History of ulceration: Range 5%–49% 3. San Antonio consensus evaluation: 58% 4. Vibration threshold with biothesiometer: 53% 5. Detailed neurological assessment: 70% 6. Hoffmann's reflex test: 0%	SWME vs. reference test: 1. Nerve conduction study: Range: 0% (95% CI: 0%–37%) to 25% (95% CI: 16%–36%) 2. History of ulceration: Range 15–35 3. San Antonio consensus evaluation: 2 4. Vibration threshold with biothesiometer: 3 5. Detailed neurological assessment: 7 6. Hoffmann's reflex test: 13	NA

Hirschfeld et al., [19]	Tuning fork (3 studies): Range 0.74 (0.01–38.65) to 2.29 (0.04–120.75) Monofilament (2 studies): Range 0.43 (0.15–1.24) to 19.00 (3.61–99.94) Biothesiometer (2 studies): Range 2.86 (1.10–7.39) to 13.5 (2.78–65.5)	Tuning fork (3 studies): Age- and gender-specific reference values (1 study); NR (2 studies) Monofilament (2 studies): Age- and gender-specific reference values (1 study); inability to sense the 4.17-U filament (1 study) Biothesiometer (2 studies): > 97th percentile of healthy control subjects (1 study); > 0.5 *μ*m (1 study)	NR	NR	NA	NA	NA

Hu, [20]	NR	1. 10-g monofilament: NR 2. Vibration perception threshold (VPT ≥ 25 V) 3. Neuropathy disability score (> 6)	PPV of IpTT vs. reference test: 10-g monofilament: Range 85%–89.9% (3 studies; 932 patients) Vibration perception threshold (VPT ≥ 25 V):29%–98.2% (3 studies; 699 patients) Neuropathy disability score (> 6): 79%–94.5% (2 studies; 434 studies)	NPV of IpTT vs. reference test: 10-g monofilament: Range 90%–97.21% (3 studies; 932 patients) Vibration perception threshold (VPT ≥ 25 V):77%–100% (3 studies; 699 patients) Neuropathy disability score (> 6): 91%–100% (2 studies; 434 studies)	1–*S* of IpTT vs. reference test: 10-g monofilament: Range 16.7%–49% (3 studies; 932 patients) Vibration perception threshold (VPT ≥ 25 V): 0%–24% (3 studies; 699 patients) Neuropathy disability score (> 6): 0%–47% (2 studies; 434 studies)	1 − Sp of IpTT vs. reference test: 10-g monofilament: Range 2%–3.6% (3 studies; 932 patients) Vibration perception threshold (VPT ≥ 25 V):3.4%–8% (3 studies; 699 patients) Neuropathy disability score (> 6): 3%–9.7% (2 studies; 434 studies)	NA

Tsapas, [16]	Diagnostic odds ratio (DOR) of 11.3 (95% CI 5.3–24.0) vs. NDS (highest available threshold ≥ 6) 11.6 (95% CI 6.0–22.8) (3 studies) vs. DNI 5.7 (95% CI 1.5–22.1) (five studies)	• Socks and shoes are removed, followed by a 10-min acclimatization at 20°C–25°C • Plaster is placed on the sole (first–second metatarsal level) and observed for color change after 10 min • Results: Normal—Full blue-to-pink change, abnormal—Partial blue change or no change; assesses vascular health	NR	NR	14% (range 0–57, 95% CI 9–21)	35% (range 0–78, 95% CI 24–49)	*S*: 90.13% (86.7–93.5) Sp: 94.96% (93.5–96.4)

Dros et al., [17]	NR	It varies: ≥ 5 of 10 incorrect (1 foot) 1 study ≥ 5 of 8 incorrect (both feet) 1 study NR 1 study	NR	NR	Range 6.9 (1–23) to 59.1 (54–64)	Range 0 (0–37) to 5.1 (1–14)	NA

Abbreviations: DOR, diagnostic odds ratio; NPV, negative predictive value; PPV, positive predictive value.

## Data Availability

The data that support the findings of this study are available from the corresponding author upon reasonable request. Additional information of methods and results are included in the Supporting Information section.

## Supporting Information

Supporting Information Additional supporting information can be found online in the Supporting Information section. This section includes supporting methods and supporting data (Appendix S1, details of AMSTAR 2.0, supporting list of excluded full texts, and Table S1—QUADAS).

## References

[1] Reynolds L., Luo Z., Singh K. (2023). Diabetic complications and prospective immunotherapy. *Frontiers in Immunology*.

[2] Sun H., Saeedi P., Karuranga S. (2022). IDF Diabetes Atlas: Global, regional and country-level diabetes prevalence estimates for 2021 and projections for 2045. *Diabetes Research and Clinical Practice*.

[3] Deshpande A. D., Harris-Hayes M., Schootman M. (2008). Epidemiology of diabetes and diabetes-related complications. *Physical therapy*.

[4] Bondar A. C., Popa A. R. (2018). Diabetic neuropathy prevalence and its associated risk factors in two representative groups of type 1 and type 2 diabetes mellitus patients from Bihor County. *Mædica*.

[5] Boulton A. J. M., Knight G., Drury J., Ward J. D. (1985). The prevalence of symptomatic, diabetic neuropathy in an insulin-treated population. *Diabetes Care*.

[6] Dyck P. J., Kratz K. M., Karnes J. L. (1993). The prevalence by staged severity of various types of diabetic neuropathy, retinopathy, and nephropathy in a population-based cohort. *Neurology*.

[7] Feldman E. L., Callaghan B. C., Pop-Busui R. (2019). Diabetic neuropathy. *Nature Reviews Disease Primers*.

[8] Ziegler D., Tesfaye S., Spallone V. (2022). Screening, diagnosis and management of diabetic sensorimotor polyneuropathy in clinical practice: international expert consensus recommendations. *Diabetes Research and Clinical Practice*.

[9] Carmichael J., Fadavi H., Ishibashi F., Shore A. C., Tavakoli M. (2021). Advances in screening, early diagnosis and accurate staging of diabetic neuropathy. *Frontiers in Endocrinology*.

[10] Selvarajah D., Kar D., Khunti K. (2019). Diabetic peripheral neuropathy: advances in diagnosis and strategies for screening and early intervention. *The Lancet Diabetes & Endocrinology*.

[11] Bönhof G. J., Herder C., Ziegler D. (2022). Diagnostic tools, biomarkers, and treatments in diabetic polyneuropathy and cardiovascular autonomic neuropathy. *Current Diabetes Reviews*.

[12] Chapter V: Overviews of reviews. https://training.cochrane.org/handbook/current/chapter-v.

[13] Whiting P., Savović J., Higgins J. P. T. (2016). ROBIS: a new tool to assess risk of bias in systematic reviews was developed. *Journal of Clinical Epidemiology*.

[14] Shea B. J., Reeves B. C., Wells G. (2017). AMSTAR 2: a critical appraisal tool for systematic reviews that include randomised or non-randomised studies of healthcare interventions, or both. *BMJ*.

[15] Zhao N., Xu J., Zhou Q. (2021). Application of the Ipswich touch test for diabetic peripheral neuropathy screening: a systematic review and meta-analysis. *BMJ Open*.

[16] Tsapas A., Liakos A., Paschos P. (2014). A simple plaster for screening for diabetic neuropathy: a diagnostic test accuracy systematic review and meta-analysis. *Metabolism*.

[17] Dros J., Wewerinke A., Bindels P. J., van Weert H. C. (2009). Accuracy of monofilament testing to diagnose peripheral neuropathy: a systematic review. *The Annals of Family Medicine*.

[18] Feng Y., Schlösser F. J., Sumpio B. E. (2009). The Semmes Weinstein monofilament examination as a screening tool for diabetic peripheral neuropathy. *Journal of Vascular Surgery*.

[19] Hirschfeld G., von Glischinski M., Blankenburg M., Zernikow B. (2014). Screening for peripheral neuropathies in children with diabetes: a systematic review. *Pediatrics*.

[20] Hu A., Koh B., Teo M. R. (2021). A review of the current evidence on the sensitivity and specificity of the Ipswich touch test for the screening of loss of protective sensation in patients with diabetes mellitus. *Diabetology International*.

[21] Wang F., Zhang J., Yu J. (2017). Diagnostic accuracy of monofilament tests for detecting diabetic peripheral neuropathy: a systematic review and meta-analysis. *Journal of Diabetes Research*.

[22] Burda B. U., Holmer H. K., Norris S. L. (2016). Limitations of a measurement tool to assess systematic reviews (AMSTAR) and suggestions for improvement. *Systematic Reviews*.

[23] Bae J. M. (2014). An overview of systematic reviews of diagnostic tests accuracy. *Epidemiology and Health*.

[24] Glas A. S., Lijmer J. G., Prins M. H., Bonsel G. J., Bossuyt P. M. M. (2003). The diagnostic odds ratio: a single indicator of test performance. *Journal of Clinical Epidemiology*.

[25] Yang B., Olsen M., Vali Y. (2021). Study designs for comparative diagnostic test accuracy: a methodological review and classification scheme. *Journal of Clinical Epidemiology*.

[26] Dube S., Hulke S. M., Wakode S. L. (2022). Effectiveness of Semmes Weinstein 10 gm monofilament in diabetic peripheral neuropathy taking nerve conduction and autonomic function study as reference tests. *Journal of Family Medicine and Primary Care*.

[27] Booth J., Young M. J. (2000). Differences in the performance of commercially available 10-g monofilaments. *Diabetes Care*.

[28] Rayman G., Vas P. R., Baker N. (2011). The Ipswich touch test. *Diabetes Care*.

[29] International Working Group on the Diabetic Foot (IWGDF) (2023). *Prevention guideline (2023 update)*.

[30] Papanas N., Giassakis G., Papatheodorou K. (2007). Sensitivity and specificity of a new indicator test (Neuropad) for the diagnosis of peripheral neuropathy in type 2 diabetes patients: a comparison with clinical examination and nerve conduction study. *Journal of Diabetes and its Complications*.

[31] Buchmann S. J., Penzlin A. I., Kubasch M. L., Illigens B. M. W., Siepmann T. (2019). Assessment of sudomotor function. *Clinical Autonomic Research*.

[32] Casellini C. M., Parson H. K., Richardson M. S., Nevoret M. L., Vinik A. I. (2013). Sudoscan, a noninvasive tool for detecting diabetic small fiber neuropathy and autonomic dysfunction. *Diabetes Technology & Therapeutics*.

[33] Xiong Q., Lu B., Ye H., Wu X., Zhang T., Li Y. (2016). The diagnostic value of neuropathy symptom and change score, neuropathy impairment score and Michigan Neuropathy Screening Instrument for diabetic peripheral neuropathy. *European Neurology*.

[34] Dong B., Lyu G., Yang X., Wang H., Chen Y. (2022). Shear wave elastography as a quantitative biomarker of diabetic peripheral neuropathy: a systematic review and meta-analysis. *Frontiers Public Health*.

[35] Chen Y., Duan H., Huang L., Jiang Z., Huang H. (2022). Supersonic shear wave imaging of the tibial nerve for diagnosis of diabetic peripheral neuropathy: a meta-analysis. *Frontiers in Endocrinology*.

[36] Dobrow M. J., Hagens V., Chafe R., Sullivan T., Rabeneck L. (2018). Consolidated principles for screening based on a systematic review and consensus process. *Canadian Medical Association Journal*.

[37] Galosi E., Hu X., Michael N., Nyengaard J. R., Truini A., Karlsson P. (2022). Redefining distal symmetrical polyneuropathy features in type 1 diabetes: a systematic review. *Acta Diabetologica*.

[38] Gad H., Petropoulos I. N., Khan A. (2022). Corneal confocal microscopy for the diagnosis of diabetic peripheral neuropathy: a systematic review and meta-analysis. *Journal of Diabetes Investigation*.

[39] Malik R. A., Kallinikos P., Abbott C. A. (2003). Corneal confocal microscopy: a non-invasive surrogate of nerve fibre damage and repair in diabetic patients. *Diabetologia*.

[40] Ahmed A., Bril V., Orszag A. (2012). Detection of diabetic sensorimotor polyneuropathy by corneal confocal microscopy in type 1 diabetes. *Diabetes Care*.

[41] Oh T. J., Song Y., Jang H. C., Choi S. H. (2022). SUDOSCAN in combination with the Michigan Neuropathy Screening Instrument is an effective tool for screening diabetic peripheral neuropathy. *Diabetes & Metabolism Journal*.

[42] Ma X., Li M., Xie H. (2024). Ankle reflex and neurological symptom score: a primary level screening method for diabetic peripheral neuropathy. *Endocrine Journal*.

[43] Qian Y., Alhaskawi A., Dong Y., Ni J., Abdalbary S., Lu H. (2024). Transforming medicine: artificial intelligence integration in the peripheral nervous system. *Frontiers in Neurology*.

[44] Gauthier-Umaña C., Valderrama M., Múnera A., Nava-Mesa M. O. (2023). BOARD-FTD-PACC: a graphical user interface for the synaptic and cross-frequency analysis derived from neural signals. *Brain Informatics*.

[45] Park D., Kim B. H., Lee S. E. (2021). Machine learning-based approach for disease severity classification of carpal tunnel syndrome. *Scientific Reports*.

[46] Matsuda K., Han X., Matsuda N., Yamanaka M., Suzuki I. (2023). Development of an in vitro assessment method for chemotherapy-induced peripheral neuropathy (CIPN) by integrating a microphysiological system (MPS) with morphological deep learning of soma and axonal images. *Toxics*.

