# Supplementary material for: Diagnostic Accuracy of Screening Tests for Diabetic Peripheral Neuropathy: An Umbrella Review
Source: J Diabetes Res. 2024 Dec 4;2024:5902036. doi: 10.1155/jdr/5902036 (PMC11634407; doi:10.1155/jdr/5902036)
Supplement: Supporting Information — Additional supporting information can be found online in the Supporting Information section. This section includes supporting methods and supporting data (Appendix S1, details of AMSTAR 2.0, supporting list of excluded full texts, and Table S1—QUADAS). [file 5902036.f1.zip › Appendix 1 Search Strategy.pdf]

SEARCH SR MA

Search electronic report #1	
**Search type**	New
**Databases**	▪ Ovid MEDLINE(R) ALL <1946 to April 03, 2023>
**Platform**	Ovid.com
**Search date**	04/04/2023
**Update date**	Undefined
**Range of search date**	None
**Language restrictions**	None
**Other limits**	Ovid Filter for SR (maximizes specificity)
**Search strategy (results)**	1 exp Diabetic Neuropathies/ (26345)<br>2 (diabet$ adj5 (neuropath$ or mononeur$ or polyneur$ or neuritis or neuralgia$ or amyotrophy$)).tw. (18242)<br>3 exp Mass Screening/ (143256)<br>4 screening$.tw. (654554)<br>5 prescreening.tw. (1749)<br>6 exp Early Diagnosis/ (66548)<br>7 (early adj5 (diagnosis or detection)).tw. (216998)<br>8 1 or 2 (33521)<br>9 3 or 4 or 5 or 6 or 7 (905541)<br>10 8 and 9 (2114)<br>11 ((achilles or ankle) adj5 reflex adj5 test$).tw. (43)<br>12 pinprick.tw. (1679)<br>13 exp Thermosensing/ (3107)<br>14 (temperature adj5 (sens* or perception)).tw. (38407)<br>15 (thermoperception or thermosens$).tw. (7700)<br>16 'tip therm'.tw. (5)<br>17 (aesthesiometer$ or 'esthesiometer' or 'pinwheel aesthesiometer' or 'pinwheel esthesiometer' or 'point aesthesiometer' or 'point esthesiometer' or 'Pressure-Specified Sensory Device' or 'Semmes-Weinstein' or 'neurosensory testing device' or 'VibraTip' or 'Vibratron II' or 'neurosensory testing device' or monofilament$).tw. (4685)<br>18 Ipswich.tw. (197)<br>19 Iptt.tw. (29)<br>20 (Von adj5 Frey$).tw. (3882)<br>21 (tuning adj5 fork).tw. (840)<br>22 ("128" adj5 Hz).tw. (321)<br>23 exp Valsalva Maneuver/ (4301)<br>24 Valsalva$.tw. (10796)<br>25 ('heart rate' adj5 variability).tw. (23696)<br>26 (deep adj5 breath$).tw. (3804)<br>27 exp Breath Tests/ (16238)<br>28 (breath$ adj5 (test$ or analys$)).tw. (15746)<br>29 (postural adj5 blood adj5 pressure adj5 test$).tw. (30)<br>30 (sweat$ adj5 test$).tw. (1757)<br>31 (silicon adj5 impression adj5 mold adj5 technique).tw. (1)<br>32 (Steel adj5 'ball bearing').tw. (27)<br>33 'jendrassik'.tw. (184)<br>34 (Sympathetic adj5 skin adj5 response).tw. (749)

	35 exp Point-of-Care Testing/ (4070)<br>36 'point of care'.tw. (30540)<br>37 (bedside adj5 test$).tw. (2113)<br>38 POCT.tw. (2709)<br>39 exp Nerve Conduction Studies/ (9)<br>40 electroneurograph$.tw. (884)<br>41 (nerve adj5 conduct$ adj5 (test$ or stud$ or device$)).tw. (7955)<br>42 (neuroconduction adj5 (test$ or stud$ or device$)).tw. (7)<br>43 DPN$.tw. (4321)<br>44 (NC adj5 Stat).tw. (33)<br>45 (Indicator adj5 plaster adj5 method).tw. (1)<br>46 (sudomotor adj5 function).tw. (372)<br>47 neuropad.tw. (50)<br>48 (galvanic adj5 skin adj5 response).tw. (692)<br>49 Sudoscan.tw. (126)<br>50 (skin adj5 conduct$ adj5 (sensor or meter)).tw. (20)<br>51 (corneal adj5 confocal adj5 microscopy).tw. (1056)<br>52 neuropen.tw. (10)<br>53 (Tiptherm or Tip-therm).tw. (8)<br>54 NerveCheck.tw. (4)<br>55 (quantitative adj5 sens$ adj5 test$).tw. (3018)<br>56 (TQST or QST).tw. (1384)<br>57 TSA$.tw. (13180)<br>58 (NeuroSensory adj5 Analyzer).tw. (11)<br>59 VibraTip.tw. (14)<br>60 (LDIflare or LDI or (laser adj5 Doppler adj5 imag$)).tw. (2790)<br>61 (flare adj5 response).tw. (588)<br>62 (sensor$ adj5 test$).tw. (13692)<br>63 (Tactile adj5 circumferential adj5 discriminator).tw. (6)<br>64 algometer.tw. (835)<br>65 neurotip.tw. (11)<br>66 neuroQuick.tw. (6)<br>67 (vibrati$ adj5 (sens$ or perception)).tw. (4617)<br>68 biothesiometer.tw. (124)<br>69 vibrameter.tw. (24)<br>70 Neurothesiometer.tw. (56)<br>71 Maxivibrometer.tw. (1)<br>72 Vibratron.tw. (22)<br>73 (CASE adj5 IV).tw. (6285)<br>74 dn4.tw. (580)<br>75 (Douleur adj5 Neuropathique adj5 Question$).tw. (234)<br>76 (michigan adj5 neuropathy adj5 screening adj5 instrument).tw. (263)<br>77 MNSI$.tw. (357)<br>78 norfolk$.tw. (1719)<br>79 (neuro$ adj5 symptom adj5 score).tw. (355)<br>80 NSS.tw. (4302)<br>81 dns.tw. (2049)<br>82 (neuropathy adj5 symptom$ adj5 profile).tw. (18)<br>83 NSP.tw. (2917)

	84 (Clinical adj5 Neurological adj5 Exam$).tw. (1482)<br>85 CNE.tw. (1267)<br>86 (Neuropathy adj5 Disability adj5 Score).tw. (292)<br>87 NDS.tw. (2834)<br>88 (Diabetic adj5 Neuropathy adj5 Exam$).tw. (195)<br>89 DNE.tw. (169)<br>90 'neuropathy impairment score'.tw. (131)<br>91 NIS$.tw. (29735)<br>92 (Michigan adj5 Diabet$).tw. (163)<br>93 MDNS.tw. (56)<br>94 'utah early neuropathy scale'.tw. (19)<br>95 UENS.tw. (23)<br>96 (leeds adj5 neuro$).tw. (284)<br>97 sLANSS.tw. (3)<br>98 LANSS.tw. (248)<br>99 (Toronto adj5 Clinic$).tw. (756)<br>100 TCSS.tw. (673)<br>101 TCNS.tw. (86)<br>102 mTCNS.tw. (18)<br>103 (neuropathic adj5 pain adj5 question$).tw. (378)<br>104 NPQ.tw. (1564)<br>105 (Neuropathic adj5 Pain adj5 Symptoms adj5 Inventory).tw. (27)<br>106 NPSI.tw. (174)<br>107 Neuro-QOL-28.tw. (0)<br>108 (Neuro$ adj5 QOL$).tw. (833)<br>109 painDETECT.tw. (411)<br>110 'numeric rating scale'.tw. (5526)<br>111 NRS.tw. (11512)<br>112 exp Visual Analog Scale/ (4100)<br>113 (Visual adj5 Analog adj5 Scale).tw. (35598)<br>114 VAS.tw. (61265)<br>115 (Diabetes adj5 Bowel adj5 Symptom adj5 Question$).tw. (4)<br>116 11 or 12 or 13 or 14 or 15 or 16 or 17 or 18 or 19 or 20 or 21 or 22 or 23 or 24 or 25 or 26 or 27 or 28 or 29 or 30 or 31 or 32 or 33 or 34 or 35 or 36 or 37 or 38 or 39 or 40 or 41 or 42 or 43 or 44 or 45 or 46 or 47 or 48 or 49 or 50 or 51 or 52 or 53 or 54 or 55 or 56 or 57 or 58 or 59 or 60 or 61 or 62 or 63 or 64 or 65 or 66 or 67 or 68 or 69 or 70 or 71 or 72 or 73 or 74 or 75 or 76 or 77 or 78 or 79 or 80 or 81 or 82 or 83 or 84 or 85 or 86 or 87 or 88 or 89 or 90 or 91 or 92 or 93 or 94 or 95 or 96 or 97 or 98 or 99 or 100 or 101 or 102 or 103 or 104 or 105 or 106 or 107 or 108 or 109 or 110 or 111 or 112 or 113 or 114 or 115 (353629)<br>117 10 and 116 (959)<br>118 limit 117 to "reviews (maximizes specificity)" (33)
**# of records identified**	33

Search electronic report #2	
**Search type**	New
**Databases**	▪ Embase
**Platform**	Embase.com
**Search date**	04/04/2023
**Update date**	Undefined
**Range of search date**	None
**Language restrictions**	None
**Other limits**	Embase filter for SR
**Search strategy (results)**	#1. 'diabetic neuropathy'/syn 33,150<br>#2. (diabet* NEAR/5 (neuropath* OR mononeur* OR polyneur* OR neuritis OR neuralgia* OR amyotroph*)):ab,ti 27,265<br>#3. #1 OR #2 40,087<br>#4. 'screening'/exp 803,304<br>#5. screening*:ab,ti 919,481<br>#6. prescreening:ab,ti 4,345<br>#7. 'early diagnosis'/exp 125,398<br>#8. (early NEAR/5 (diagnosis OR detection)):ab,ti 314,351<br>#9. #4 OR #5 OR #6 OR #7 OR #8 1,599,282<br>#10. #3 AND #9 3,265<br>#11. ((achilles OR ankle) NEAR/5 reflex NEAR/5 test*):ab,ti 59<br>#12. 'pinprick test'/exp 1,027<br>#13. pinprick:ab,ti 3,073<br>#14. 'temperature sense'/exp 6,134<br>#15. (temperature NEAR/5 (sens* OR perception)):ab,ti 38,164<br>#16. thermoperception:ab,ti OR thermosens*:ab,ti 9,483<br>#17. 'tip-therm':ab,ti 13<br>#18. 'neurosensory testing device'/exp 486<br>#19. aesthesiometer*:ab,ti OR 'esthesiometer':ab,ti OR 'pinwheel aesthesiometer':ab,ti OR 'pinwheel esthesiometer':ab,ti OR 'point aesthesiometer':ab,ti OR 'point esthesiometer':ab,ti OR 'pressure-specified sensory device':ab,ti OR 'semmes-weinstein':ab,ti OR 'vibratip':ab,ti OR 'vibratron ii':ab,ti OR 'neurosensory testing device':ab,ti OR monofilament*:ab,ti 6,724<br>#20. 'ipswich touch test':ab,ti 29<br>#21. iptt:ab,ti 52<br>#22. 'von frey test'/exp 2,014<br>#23. (von NEAR/5 frey*):ab,ti 5,894<br>#24. 'tuning fork'/exp 633<br>#25. '128 hz':ab,ti OR 'tuning fork':ab,ti 1,103<br>#26. 'valsalva maneuver'/exp 10,466<br>#27. valsalva*:ab,ti 16,516<br>#28. 'heart rate variability'/exp 33,785<br>#29. ('heart rate' NEAR/5 variability):ab,ti 33,083<br>#30. 'deep breathing test'/exp 40<br>#31. (deep NEAR/5 breath*):ab,ti 6,763

	#32. 'breath analysis'/exp 22,381<br>#33. (breath* NEAR/5 (test* OR analys*)):ab,ti 24,158<br>#34. (postural NEAR/5 blood NEAR/5 pressure NEAR/5 test*):ab,ti 45<br>#35. 'sweat test'/exp 2,850<br>#36. (sweat* NEAR/5 test*):ab,ti 3,414<br>#37. (silicon NEAR/5 impression NEAR/5 mold NEAR/5 technique):ab,ti 1<br>#38. (steel NEAR/5 'ball-bearing'):ab,ti 5<br>#39. jendrassik:ab,ti 264<br>#40. (sympathetic NEAR/5 skin NEAR/5 response):ab,ti 1,174<br>#41. 'point of care testing'/exp 20,578<br>#42. 'point of care':ab,ti 40,688<br>#43. (bedside NEAR/5 test*):ab,ti 3,290<br>#44. poct:ab,ti 3,513<br>#45. 'electroneurography'/exp 3,202<br>#46. electroneurograph*:ab,ti 1,264<br>#47. (nerve NEAR/5 conduct* NEAR/5 (test* OR stud* OR 13,737 device*)):ab,ti<br>#48. (neuroconduction NEAR/5 (test* OR stud* OR device*)):ab,ti 23<br>#49. 'dpn-check':ab,ti OR dpncheck:ab,ti 45<br>#50. 'nc-stat':ab,ti 38<br>#51. (indicator NEAR/5 plaster NEAR/5 method):ab,ti 2<br>#52. 'sudomotor function measurement device'/exp 355<br>#53. (sudomotor NEAR/5 function):ab,ti 675<br>#54. neuropad:ab,ti 86<br>#55. 'galvanic skin response sensor'/exp 328<br>#56. (galvanic NEAR/5 skin NEAR/5 response):ab,ti 914<br>#57. sudoscan:ab,ti 294<br>#58. (skin NEAR/5 conduct* NEAR/5 (sensor OR meter)):ab,ti 31<br>#59. 'corneal confocal microscopy'/exp 110<br>#60. (corneal NEAR/5 confocal NEAR/5 microscopy):ab,ti 1,619<br>#61. neuropen:ab,ti 23<br>#62. tiptherm:ab,ti OR 'tip therm':ab,ti 10<br>#63. nervecheck:ab,ti 7<br>#64. 'quantitative sensory testing'/exp 1,282<br>#65. (quantitative NEAR/5 sens* NEAR/5 test*):ab,ti 4,316<br>#66. tqst:ab,ti OR qst:ab,ti 2,168<br>#67. 'tsa-ii':ab,ti OR 'neurosensory analyzer':ab,ti 82<br>#68. vibratip:ab,ti 24<br>#69. ldiflare:ab,ti OR ldi:ab,ti OR ((laser NEAR/5 doppler NEAR/5 imag*):ab,ti) 3,861<br>#70. (flare NEAR/5 response):ab,ti 936<br>#71. 'sensory perception test'/exp 388<br>#72. (sensor* NEAR/5 test*):ab,ti 17,356<br>#73. (tactile NEAR/5 circumferential NEAR/5 discriminator):ab,ti 6<br>#74. 'algometer'/exp 1,062

	#75. algometer:ab,ti	1,282
	#76. neurotip:ab,ti	16
	#77. 'vibration sense'/exp	3,705
	#78. neuroquick:ab,ti	7
	#79. (vibrati* NEAR/5 (sens* OR perception)):ab,ti	5,694
	#80. 'biothesiometer'/exp	114
	#81. biothesiometer:ab,ti	238
	#82. vibrameter:ab,ti	27
	#83. neurothesiometer:ab,ti	99
	#84. maxivibrometer:ab,ti	1
	#85. vibratron:ab,ti	40
	#86. ('case iv' NEAR/5 (system* OR test* OR sens*)):ab,ti	32
	#87. 'dn4 questionnaire'/exp	33
	#88. 'dn4':ab,ti	1,238
	#89. (douleur NEAR/5 neuropathique NEAR/5 question*):ab,ti	323
	#90. 'michigan neuropathy screening instrument'/exp	95
	#91. 'michigan neuropathy screening instrument questionnaire'/exp	19
	#92. (michigan NEAR/5 neuropathy NEAR/5 screening):ab,ti	459
	#93. mnsi*:ab,ti	497
	#94. 'norfolk quality of life diabetic neuropathy questionnaire'/exp	14
	#95. norfolk*:ab,ti	2,759
	#96. 'neuropathy symptom score'/exp	49
	#97. (neuro* NEAR/5 symptom NEAR/5 score):ab,ti	610
	#98. nss:ab,ti	5,859
	#99. 'diabetic neuropathy symptom score'/exp	19
	#100.dns:ab,ti	2,888
	#101.(neuropathy NEAR/5 symptom* NEAR/5 profile):ab,ti	43
	#102.nsp:ab,ti	3,237
	#103.(clinic* NEAR/5 neurolog* NEAR/5 exam*):ab,ti	4,255
	#104.cne:ab,ti	1,707
	#105.'neuropathy disability score'/exp	109
	#106.(neuropathy NEAR/5 disability NEAR/5 score):ab,ti	540
	#107.nds:ab,ti	3,649
	#108.(diabetic NEAR/5 neuropathy NEAR/5 examination):ab,ti	99
	#109.dne:ab,ti	292
	#110.'neuropathy impairment score'/exp	47
	#111.'neuropathy impairment score':ab,ti	343
	#112.nis:ab,ti	14,793
	#113.'nis ll':ab,ti	173
	#114.'michigan diabetic neuropathy scale':ab,ti	5
	#115.mdns:ab,ti	94
	#116.'utah early neuropathy scale'/exp	17
	#117.'utah early neuropathy scale':ab,ti	41
	#118.uens:ab,ti	70
	#119.'leeds assessment of neuropathic symptoms and	64

	signs'/exp<br>#120.'leeds assessment of neuropathic symptoms and signs':ab,ti 346<br>#121.lanss:ab,ti 452<br>#122.slanss:ab,ti 150<br>#123.'toronto clinical scoring system'/exp 36<br>#124.'toronto clinical neuropathy score'/exp 18<br>#125.'toronto clinical':ab,ti 207<br>#126.tcss:ab,ti 765<br>#127.tcns:ab,ti 133<br>#128.mtcns:ab,ti 25<br>#129.'neuropathic pain questionnaire'/exp 39<br>#130.'neuropathic pain questionnaire':ab,ti 90<br>#131.npq:ab,ti 1,464<br>#132.'neuropathic pain symptoms inventory':ab,ti 15<br>#133.npsi:ab,ti 290<br>#134.'brief pain inventory':ab,ti 4,700<br>#135.bpi:ab,ti 5,337<br>#136.'neuro qol':ab,ti 426<br>#137.'paindetect'/exp 28<br>#138.paindetect:ab,ti 767<br>#139.'numeric rating scale'/exp 17,079<br>#140.'numeric rating scale':ab,ti 8,321<br>#141.nrs:ab,ti 18,291<br>#142.'visual analog scale'/exp 117,698<br>#143.'visual analog scale':ab,ti 44,086<br>#144.vas:ab,ti 99,916<br>#145.'diabetes bowel symptom questionnaire':ab,ti 5<br>#146.#11 OR #12 OR #13 OR #14 OR #15 OR #16 OR #17 OR 513,166<br>#18 OR #19 OR #20 OR #21 OR #22 OR #23 OR #24 OR #25 OR #26 OR #27 OR #28 OR #29 OR #30 OR #31 OR #32 OR #33 OR #34 OR #35 OR #36 OR #37 OR #38 OR #39 OR #40 OR #41 OR #42 OR #43 OR #44 OR #45 OR #46 OR #47 OR #48 OR #49 OR #50 OR #51 OR #52 OR #53 OR #54 OR #55 OR #56 OR #57 OR #58 OR #59 OR #60 OR #61 OR #62 OR #63 OR #64 OR #65 OR #66 OR #67 OR #68 OR #69 OR #70 OR #71 OR #72 OR #73 OR #74 OR #75 OR #76 OR #77 OR #78 OR #79 OR #80 OR #81 OR #82 OR #83 OR #84 OR #85 OR #86 OR #87 OR #88 OR #89 OR #90 OR #91 OR #92 OR #93 OR #94 OR #95 OR #96 OR #97 OR #98 OR #99 OR #100 OR #101 OR #102 OR #103 OR #104 OR #105 OR #106 OR #107 OR #108 OR #109 OR #110 OR #111 OR #112 OR #113 OR #114 OR #115 OR #116 OR #117 OR #118 OR #119 OR #120 OR #121 OR #122 OR #123 OR #124 OR #125 OR #126 OR #127 OR #128 OR #129 OR #130 OR #131 OR #132 OR #133 OR #134 OR #135 OR #136 OR #137 OR #138 OR #139 OR #140 OR #141 OR #142 OR #143 OR #144 OR #145<br>#147.#10 AND #146 1,466

	#148.#10 AND #146 AND ([cochrane review]/lim OR [systematic review]/lim OR [meta analysis]/lim)	29
**# of records identified**	29	

Search electronic report #3	
**Search type**	New
**Databases**	▪ EBM Reviews - Cochrane Database of Systematic Reviews <2005 to March 28, 2023>
**Platform**	Ovid.com
**Search date**	04/04/2023
**Update date**	Undefined
**Range of search date**	None
**Language restrictions**	None
**Other limits**	None
**Search strategy (results)**	1 [exp Diabetic Neuropathies/] (0)<br>2 (diabet$ adj5 (neuropath$ or mononeur$ or polyneur$ or neuritis or neuralgia$ or amyotrophy$)).tw. (194)<br>3 [exp Mass Screening/] (0)<br>4 screening$.tw. (5122)<br>5 prescreening.tw. (8)<br>6 [exp Early Diagnosis/] (0)<br>7 (early adj5 (diagnosis or detection)).tw. (577)<br>8 1 or 2 (194)<br>9 3 or 4 or 5 or 6 or 7 (5269)<br>10 8 and 9 (87)<br>11 ((achilles or ankle) adj5 reflex adj5 test$).tw. (1)<br>12 pinprick.tw. (11)<br>13 [exp Thermosensing/] (0)<br>14 (temperature adj5 (sens* or perception)).tw. (39)<br>15 (thermoperception or thermosens$).tw. (3)<br>16 'tip therm'.tw. (0)<br>17 (aesthesiometer$ or 'esthesiometer' or 'pinwheel aesthesiometer' or 'pinwheel esthesiometer' or 'point aesthesiometer' or 'point esthesiometer' or 'Pressure-Specified Sensory Device' or 'Semmes-Weinstein' or 'neurosensory testing device' or 'VibraTip' or 'Vibratron II' or 'neurosensory testing device' or monofilament$).tw. (40)<br>18 Ipswich.tw. (8)<br>19 Iptt.tw. (0)<br>20 (Von adj5 Frey$).tw. (5)<br>21 (tuning adj5 fork).tw. (1)<br>22 ("128" adj5 Hz).tw. (1)<br>23 [exp Valsalva Maneuver/] (0)<br>24 Valsalva$.tw. (28)<br>25 ('heart rate' adj5 variability).tw. (62)<br>26 (deep adj5 breath$).tw. (57)<br>27 [exp Breath Tests/] (0)<br>28 (breath$ adj5 (test$ or analys$)).tw. (107)

	29 (postural adj5 blood adj5 pressure adj5 test$).tw. (0)<br>30 (sweat$ adj5 test$).tw. (98)<br>31 (silicon adj5 impression adj5 mold adj5 technique).tw. (0)<br>32 (Steel adj5 'ball bearing').tw. (0)<br>33 'jendrassik'.tw. (0)<br>34 (Sympathetic adj5 skin adj5 response).tw. (0)<br>35 [exp Point-of-Care Testing/] (0)<br>36 'point of care'.tw. (151)<br>37 (bedside adj5 test$).tw. (26)<br>38 POCT.tw. (12)<br>39 [exp Nerve Conduction Studies/] (0)<br>40 electroneurograph$.tw. (4)<br>41 (nerve adj5 conduct$ adj5 (test$ or stud$ or device$)).tw. (50)<br>42 (neuroconduction adj5 (test$ or stud$ or device$)).tw. (0)<br>43 DPN$.tw. (14)<br>44 (NC adj5 Stat).tw. (2)<br>45 (Indicator adj5 plaster adj5 method).tw. (0)<br>46 (sudomotor adj5 function).tw. (0)<br>47 neuropad.tw. (0)<br>48 (galvanic adj5 skin adj5 response).tw. (5)<br>49 Sudoscan.tw. (1)<br>50 (skin adj5 conduct$ adj5 (sensor or meter)).tw. (0)<br>51 (corneal adj5 confocal adj5 microscopy).tw. (1)<br>52 neuropen.tw. (0)<br>53 (Tiptherm or Tip-therm).tw. (0)<br>54 NerveCheck.tw. (0)<br>55 (quantitative adj5 sens$ adj5 test$).tw. (16)<br>56 (TQST or QST).tw. (5)<br>57 TSA$.tw. (745)<br>58 (NeuroSensory adj5 Analyzer).tw. (0)<br>59 VibraTip.tw. (0)<br>60 (LDIflare or LDI or (laser adj5 Doppler adj5 imag$)).tw. (6)<br>61 (flare adj5 response).tw. (8)<br>62 (sensor$ adj5 test$).tw. (47)<br>63 (Tactile adj5 circumferential adj5 discriminator).tw. (0)<br>64 algometer.tw. (5)<br>65 neurotip.tw. (0)<br>66 neuroQuick.tw. (0)<br>67 (vibrati$ adj5 (sens$ or perception)).tw. (23)<br>68 biothesiometer.tw. (1)<br>69 vibrameter.tw. (0)<br>70 Neurothesiometer.tw. (0)<br>71 Maxivibrometer.tw. (0)<br>72 Vibratron.tw. (0)<br>73 (CASE adj5 IV).tw. (11)<br>74 dn4.tw. (2)<br>75 (Douleur adj5 Neuropathique adj5 Question$).tw. (2)<br>76 (michigan adj5 neuropathy adj5 screening adj5 instrument).tw. (3)<br>77 MNSI$.tw. (2)

	78 norfolk$.tw. (18)<br>79 (neuro$ adj5 symptom adj5 score).tw. (14)<br>80 NSS.tw. (8)<br>81 dns.tw. (3)<br>82 (neuropathy adj5 symptom$ adj5 profile).tw. (0)<br>83 NSP.tw. (6)<br>84 (Clinical adj5 Neurological adj5 Exam$).tw. (18)<br>85 CNE.tw. (2)<br>86 (Neuropathy adj5 Disability adj5 Score).tw. (11)<br>87 NDS.tw. (12)<br>88 (Diabetic adj5 Neuropathy adj5 Exam$).tw. (4)<br>89 DNE.tw. (0)<br>90 'neuropathy impairment score'.tw. (18)<br>91 NIS$.tw. (658)<br>92 (Michigan adj5 Diabet$).tw. (3)<br>93 MDNS.tw. (1)<br>94 'utah early neuropathy scale'.tw. (1)<br>95 UENS.tw. (0)<br>96 (leeds adj5 neuro$).tw. (6)<br>97 sLANSS.tw. (0)<br>98 LANSS.tw. (6)<br>99 (Toronto adj5 Clinic$).tw. (11)<br>100 TCSS.tw. (1)<br>101 TCNS.tw. (0)<br>102 mTCNS.tw. (0)<br>103 (neuropathic adj5 pain adj5 question$).tw. (9)<br>104 NPQ.tw. (1)<br>105 (Neuropathic adj5 Pain adj5 Symptoms adj5 Inventory).tw. (0)<br>106 NPSI.tw. (3)<br>107 Neuro-QOL-28.tw. (0)<br>108 (Neuro$ adj5 QOL$).tw. (16)<br>109 painDETECT.tw. (2)<br>110 'numeric rating scale'.tw. (109)<br>111 NRS.tw. (377)<br>112 [exp Visual Analog Scale/] (0)<br>113 (Visual adj5 Analog adj5 Scale).tw. (122)<br>114 VAS.tw. (1283)<br>115 (Diabetes adj5 Bowel adj5 Symptom adj5 Question$).tw. (0)<br>116 11 or 12 or 13 or 14 or 15 or 16 or 17 or 18 or 19 or 20 or 21 or 22 or 23 or 24 or 25 or 26 or 27 or 28 or 29 or 30 or 31 or 32 or 33 or 34 or 35 or 36 or 37 or 38 or 39 or 40 or 41 or 42 or 43 or 44 or 45 or 46 or 47 or 48 or 49 or 50 or 51 or 52 or 53 or 54 or 55 or 56 or 57 or 58 or 59 or 60 or 61 or 62 or 63 or 64 or 65 or 66 or 67 or 68 or 69 or 70 or 71 or 72 or 73 or 74 or 75 or 76 or 77 or 78 or 79 or 80 or 81 or 82 or 83 or 84 or 85 or 86 or 87 or 88 or 89 or 90 or 91 or 92 or 93 or 94 or 95 or 96 or 97 or 98 or 99 or 100 or 101 or 102 or 103 or 104 or 105 or 106 or 107 or 108 or 109 or 110 or 111 or 112 or 113 or 114 or 115 (3150)

	117 10 and 116 (65)
**# of records identified**	65

**Search electronic report #4**	
**Search type**	New
**Databases**	▪ LILACS
**Platform**	iAHx - VHL Regional Portal
**Search date**	06/04/2023
**Update date**	Undefined
**Range of search date**	None
**Language restrictions**	None
**Other limits**	LILACS/SYSTEMATIC REVIEWS
**Search strategy (results)**	(diabetic neuropathy) AND (screening) AND (db:("LILACS") AND type_of_study:("systematic_reviews"))
**# of records identified**	1

**EMBASE**	**29**
**MEDLINE**	**33**
**COCHRANE**	**65**
**LILACS**	**1**
**TOTAL**	**128**

WITHOUT DUPLICATES 113
