# Supplementary material for: Diagnostic Accuracy of Screening Tests for Diabetic Peripheral Neuropathy: An Umbrella Review
Source: J Diabetes Res. 2024 Dec 4;2024:5902036. doi: 10.1155/jdr/5902036 (PMC11634407; doi:10.1155/jdr/5902036)
Supplement: Supporting Information — Additional supporting information can be found online in the Supporting Information section. This section includes supporting methods and supporting data (Appendix S1, details of AMSTAR 2.0, supporting list of excluded full texts, and Table S1—QUADAS). [file 5902036.f1.zip › Supplementary 1 List of excluded full texts.docx]

1. EZSCAN for undiagnosed type 2 diabetes mellitus: A systematic review and meta-analysis, Bernabe-Ortiz A, Ruiz-Alejos A, Miranda JJ, Mathur R, Perel P, Smeeth L The objective of the study is NOT evaluation of screening methods accuracy and diagnostic value
2. Effectiveness of bedside investigations to diagnose peripheral artery disease among people with diabetes mellitus: a systematic review, Brownrigg JRW, Hinchliffe RJ, Apelqvist J, Boyko EJ, Fitridge R, Mills JL, Reekers J, Shearman CP, Zierler RE, Schaper NC Covers topics other than screening methods performance for DPN
3. Corneal confocal microscopy for the diagnosis of diabetic sensorimotor polyneuropathy in people with type 1 and 2 diabetes mellitus, Burgess J, Petropoulos I, Gad H, Nevitt SJ, Ponirakis G, Ferdousi M, Kalteniece A, Azmi S, Kaye S, Malik RA, Alam U This is a protocol.
4. Glycated Hemoglobin (HbA1c) as a Biomarker for Diabetic Foot Peripheral Neuropathy, Casadei G, Filippini M, Brognara L, Covers topics other than screening methods performance for DPN
5. Supersonic shear wave imaging of the tibial nerve for diagnosis of diabetic peripheral neuropathy: A meta-analysis, Chen Y, Duan H, Huang L, Jiang Z, Huang H, Covers topics other than screening methods performance for DPN
6. Shear wave elastography as a quantitative biomarker of diabetic peripheral neuropathy: A systematic review and meta-analysis, Dong B, Lyu G, Yang X, Wang H, Chen Y, Covers topics other than screening methods performance for DPN
7. Point prevalence of painful diabetic neuropathy in the middle east and north africa: A systematic review with meta-analysis, Garoushi S, Tashani OA, Johnson MI, Covers topics other than screening methods performance for DPN
8. Assessment scales for the diagnosis of polyneuropathy, Hanewinckel R, Ikram MA, van Doorn PA, This is not a systematic review
9. Clinical Significance of Non-invasive Skin Autofluorescence Measurement in Patients with Diabetes: A Systematic Review and Meta-analysis, Hosseini MS, Razavi Z, Ehsani AH, Firooz A, Afazeli S, Covers topics other than screening methods performance for DPN
10. Structural, functional, and symptom relations in painful distal symmetric polyneuropathies: a systematic review, Karlsson P, Hincker AM, Jensen TS, Freeman R, Haroutounian S, The objective of the study is NOT evaluation of screening methods accuracy and diagnostic value
11. Prevalence of peripheral neuropathy in pre-diabetes: A systematic review, Kirthi V, Perumbalath A, Brown E, Nevitt S, Petropoulos IN, Burgess J, Roylance R, Cuthbertson DJ, Jackson TL, Malik RA, Alam U Study in prediabetic patients, matches exclusion criteria.
12. The use of the Semmes-Weinstein monofilament and other threshold tests for preventing foot ulceration and amputation in persons with diabetes, Mayfield JA, Sugarman JR, Covers topics other than screening methods performance for DPN
13. Differences and Similarities in Neuropathy in Type 1 and 2 Diabetes: A Systematic Review, Sempere-Bigorra M, Julian-Rochina I, Cauli O, Covers topics other than screening methods performance for DPN
14. Altered pain processing in patients with type 1 and 2 diabetes: Systematic review and meta-analysis of pain detection thresholds and pain modulation mechanisms, Sierra-Silvestre E, Somerville M, Bisset L, Coppieters MW, The objective of the study is NOT evaluation of screening methods accuracy and diagnostic value
15. Preventing foot ulcers in patients with diabetes, Singh N, Armstrong DG, Lipsky BA, The objective of the study is NOT evaluation of screening methods accuracy and diagnostic value
16. Scoring systems to screen for diabetic peripheral neuropathy, Yang Z, Chen R, Zhang Y, Huang Y, Hong T, Sun F, Ji L, Zhan S, This is a protocol
17. Simple tests to screen for diabetic peripheral neuropathy, Yang Z, Zhang Y, Chen R, Huang Y, Ji L, Sun F, Hong T, Zhan S, This is a protocol
18. Electrophysiological measurements of diabetic peripheral neuropathy: A systematic review, Shabeeb D, Najafi M, Hasanzadeh G, Hadian MR, Musa AE, Shirazi A The objective of the study is NOT evaluation of screening methods accuracy and diagnostic value
19. A systematic review of the prevalence, risk factors and screening tools for autonomic and diabetic peripheral neuropathy in children, adolescents and young adults with type 1 diabetes, Franceschi R, Mozzillo E, Di Candia F, Rosanio FM, Leonardi L, Liguori A, Micheli F, Cauvin V, Franzese A, Piona CA, Marcovecchio ML, There was not enough information
20. Redefining distal symmetrical polyneuropathy features in type 1 diabetes: a systematic review, Galosi E, Hu X, Michael N, Nyengaard JR, Truini A, Karlsson P, The objective of the study is NOT evaluation of screening methods accuracy and diagnostic value
21. Performance analysis of noninvasive electrophysiological methods for the assessment of diabetic sensorimotor polyneuropathy in clinical research: a systematic review and meta-analysis with trial sequential analysis, Haque F, Reaz MBI, Ali SHM, Arsad N, Chowdhury MEH, The objective of the study is NOT evaluation of screening methods accuracy and diagnostic value
22. Reliability of recommended non-invasive chairside screening tests for diabetes-related peripheral neuropathy: A systematic review with meta-analyses, McIllhatton A, Lanting S, Lambkin D, Leigh L, Casey S, Chuter V, Not enough information
23. Magnitude of diabetic peripheral neuropathy in Saudi Arabia: a systematic review and meta-analysis, Owolabi LF, Alghamdi M, Adamu B, Taura MG, Jibo A, Almansour M, Alaklabi SN, Alghamdi MA, Alotaibi YA, Imam IA, Abdelrazak R, Rafaat A, Aliyu MH, Not enough information
24. Large fibre, small fibre and autonomic neuropathy in adolescents with type 1 diabetes: A systematic review, Rasmussen VF, Jensen TS, Tankisi H, Karlsson P, Vestergaard ET, Kristensen K, Nyengaard JR, Terkelsen AJ, Covers topics other than screening methods performance for DPN
25. The clinical use of the 10g monofilament and its limitations: a review, Tan LS, Is not a systematic review or meta-analysis
26. Painful diabetic neuropathy: epidemiology, natural history, early diagnosis, and treatment options, Veves A, Backonja M, Malik RA, Is not a systematic review or meta-analysis
27. Nonlinear methods of heart rate variability analysis in diabetes, Vitor ALR, de Souza NM, Lorenconi RMR, Pastre CM, de Abreu LC, Valenti VE, Vanderlei LCM, Covers topics other than screening methods performance for DPN
28. Corneal confocal microscopy for the diagnosis of diabetic peripheral neuropathy: A systematic review and meta-analysis, Gad H., Petropoulos I.N., Khan A., Ponirakis G., MacDonald R., Alam U., Malik R.A. There was not enough information
29. Impact of age at type 2 diabetes mellitus diagnosis on mortality and vascular complications: systematic review and meta-analyses, Nanayakkara N., Curtis A.J., Heritier S., Gadowski A.M., Pavkov M.E., Kenealy T., Owens D.R., Thomas R.L., Song S., Wong J., Chan J.C.-N., Luk A.O.-Y., Penno G., Ji L., Mohan V., Amutha A., Romero-Aroca P., Gasevic D., Magliano D.J., Teede H.J., Chalmers J., Zoungas S. Study covers topics other than screening methods for DPN
30. Economic evaluation of the nerve conduction measuring device for the diagnosis of peripheal diabetic neuropathy, Soto Molina H., Diaz-Alvarez O., Sandoval-Avila M., Dominguez V., Sinta Cortes G., Rodriguez-Mendoza M.M. It’s a conference abstract
