# Supplementary material for: Diagnostic Accuracy of Screening Tests for Diabetic Peripheral Neuropathy: An Umbrella Review
Source: J Diabetes Res. 2024 Dec 4;2024:5902036. doi: 10.1155/jdr/5902036 (PMC11634407; doi:10.1155/jdr/5902036)
Supplement: Supporting Information — Additional supporting information can be found online in the Supporting Information section. This section includes supporting methods and supporting data (Appendix S1, details of AMSTAR 2.0, supporting list of excluded full texts, and Table S1—QUADAS). [file 5902036.f1.zip › Supplementary Material AMSTAR.docx]

Supplementary AMSTAR.

In question 1, Did the research questions and inclusion criteria for the review include the components of PICO?, 6 of the seven included studies met this criterion since the Dros et al. study [1] did not include the PICO question in the opinion of the evaluators. Concerning item 2, Did the report of the review contain an explicit statement that the review methods were established prior to the conduct of the review, and did the report justify any significant deviations from the protocol? Only two studies [2] and [3] met this criterion, while the remaining six did not. Regarding question 3, Did the review authors explain their selection of the study designs for inclusion in the review? None of the studies met this criterion in the opinion of the evaluators. In relation to question 4, Did the review authors use a comprehensive literature search strategy? The evaluators' response was "yes" for the studies by Tsapas and others [2] and Zhao et al. [3], "partial yes" for the studies by Wang et al. [4], Hu et al. [5] and Dros et al. [1], and "no" for the studies by Hirschfeld et al. [6] and Feng et al. [7]. For criterion 5, Did the review authors perform study selection in duplicate? The answer was affirmative for all studies. In the case of question 6, Did the review authors perform data extraction in duplicate? In 4 studies, the answer was affirmative [2], [3], [4], [6], and negative for the [1], [5], [7]. Regarding question 7, Did the review authors provide a list of excluded studies and justify the exclusions? The response obtained was "no" for all studies. Regarding question 8, Did the review authors describe the included studies in adequate detail? The following was found: "partial yes" for the studies by Tsapas et al. [2], Wang et al. [4], Hirschfeld et al. [6] and Dros and others [1], and "no" for the remaining studies [3], [5], and [7]. Question 9: "Did the review authors use a satisfactory technique for assessing the risk of bias (RoB) in individual studies that were included in the review?" was subdivided into two questions in such a way that for subquestion 9.1, the answer was affirmative only for the Zhao et al. study [3], while that the question was considered not applicable to the other studies. Regarding subquestion 9.2, it was answered affirmatively for 4 studies [2],[3], [4], [5], while the answer was "no" for the remaining studies. In the case of question 10, Did the review authors report on the sources of funding for the studies included in the review?, the answer was negative for all the studies. As for question 11, If meta-analysis was performed, did the review authors use appropriate methods for the statistical combination of results? This was subdivided into item 11.1 with a negative response for 3 studies [2], [3], [4], and "Not applicable" for the remaining studies. For subquestion 11.2, the answer was affirmative in the case of 2 studies [2], [3], negative for 1 [4] and "not applicable" for the remaining studies. In the case of question 12, If meta-analysis was performed, did the review authors assess the potential impact of RoB in individual studies on the results of the meta-analysis or other evidence synthesis? The answer was affirmative only for one study [2], negative for two studies [3], [4], and the question was considered not applicable to the remaining studies. In the case of question 13, Did the review authors account for RoB in individual studies when interpreting/discussing the results of the review? The answer was affirmative for the studies by Tsapas et al. [2], Zhao and others [3] and Wang et al. [4] and negative for all the others. Regarding question 14, Did the review authors provide a satisfactory explanation for, and discussion of, any heterogeneity observed in the results of the review? The raters' response was "yes" for four studies [2], [3], [4], [6], while it was "no" for the remaining studies. Concerning question 15, If they performed quantitative synthesis, did the review authors carry out an adequate investigation of publication bias (small study bias) and discuss its likely impact on the results of the review? The answer was affirmative for three studies [2], [3], [4], and "not applicable" for the studies of Hu et al. [5], Hirschfeld et al. [6] and Feng et al. [7]; and it was negative for the Dros et al. [1] study. Finally, for question 16, Did the review authors report any potential sources of conflict of interest, including any funding they received for conducting the review? It was considered that these sources were reported adequately by five studies [1], [2], [3], [4], [5]; while this was not the case in the remaining studies (see **Figure 2**).

[1] J. Dros, A. Wewerinke, P. J. Bindels, and H. C. van Weert, “Accuracy of Monofilament Testing to Diagnose Peripheral Neuropathy: A Systematic Review,” *Ann. Fam. Med.*, vol. 7, no. 6, pp. 555–558, Nov. 2009, doi: 10.1370/afm.1016.

[2] A. Tsapas *et al.*, “A simple plaster for screening for diabetic neuropathy: A diagnostic test accuracy systematic review and meta-analysis,” *Metab. - Clin. Exp.*, vol. 63, no. 4, pp. 584–592, Apr. 2014, doi: 10.1016/j.metabol.2013.11.019.

[3] N. Zhao *et al.*, “Application of the Ipswich Touch Test for diabetic peripheral neuropathy screening: a systematic review and meta-analysis,” *BMJ Open*, vol. 11, no. 10, p. e046966, Oct. 2021, doi: 10.1136/bmjopen-2020-046966.

[4] F. Wang *et al.*, “Diagnostic Accuracy of Monofilament Tests for Detecting Diabetic Peripheral Neuropathy: A Systematic Review and Meta-Analysis,” *J. Diabetes Res.*, vol. 2017, p. 8787261, 2017, doi: 10.1155/2017/8787261.

[5] A. Hu, B. Koh, and M.-R. Teo, “A review of the current evidence on the sensitivity and specificity of the Ipswich touch test for the screening of loss of protective sensation in patients with diabetes mellitus,” *Diabetol. Int.*, vol. 12, no. 2, pp. 145–150, Jun. 2020, doi: 10.1007/s13340-020-00451-9.

[6] G. Hirschfeld, M. von Glischinski, M. Blankenburg, and B. Zernikow, “Screening for Peripheral Neuropathies in Children With Diabetes: A Systematic Review,” *Pediatrics*, vol. 133, no. 5, pp. e1324–e1330, May 2014, doi: 10.1542/peds.2013-3645.

[7] Y. Feng, F. J. Schlösser, and B. E. Sumpio, “The Semmes Weinstein monofilament examination as a screening tool for diabetic peripheral neuropathy,” *J. Vasc. Surg.*, vol. 50, no. 3, pp. 675-682.e1, Sep. 2009, doi: 10.1016/j.jvs.2009.05.017.
