# Supplementary material for: Diagnostic Accuracy of Screening Tests for Diabetic Peripheral Neuropathy: An Umbrella Review
Source: J Diabetes Res. 2024 Dec 4;2024:5902036. doi: 10.1155/jdr/5902036 (PMC11634407; doi:10.1155/jdr/5902036)
Supplement: Supporting Information — Additional supporting information can be found online in the Supporting Information section. This section includes supporting methods and supporting data (Appendix S1, details of AMSTAR 2.0, supporting list of excluded full texts, and Table S1—QUADAS). [file 5902036.f1.zip › Supplementary Table 1 quadas(1).docx]

| Zhao 2021 | Wang 2017 | Feng 2009 | Hirschfeld 2014 | Hu 2021 | Tsapas 2014 | Dros, 2009 |
| --- | --- | --- | --- | --- | --- | --- |
| The quality of the included studies was assessed using QUADAS 1.0. Unclear risk of bias questions 4 and 14. High or unclear risk of bias question 13. High ris of bias question 9 | assessed the methodological quality of the studies using Quality Assessment of Diagnostic Accuracy Studies (QUADAS-2); Patient selection Unclear; Index test Unclear; Refrence standard low; Flow and timing Unclear | NR | Quality Assessment of Diagnostic Accuracy Studies (QUADAS-2) limitation: tests results possibly not interpreted without knowledge of the other test results (2 studies); QUADAS limitation: execution of index test and reference standard not described in sufficient detail (2 studies); QUADAS limitation: interpretation of screening and/or index results not clearly described (1 study) | Methodological quality was assessed using the QUADAS-2 tool; Low risk of bias 2 studies; Unclear risk of bias 3 studies (it was not mentioned that there was blinding of the results during the interpretation of both the index and reference tests) | We assessed methodological quality of included studies utilizing the QUADAS 2 tool; Patient selection low risk of bias (6 studies); Use of index test low risk of bias (5 studies); Use refereence standard: low risk of bias (5 studies); flow and timing low rosk of bias (13 studies) | Use of QUADAS 1; QUADAS limitation: selection criteria not clearly described (2 studies); QUADAS limitation: test results possibly not interpreted without knowledge of the other test results (1 study); QUADAS limitation: withdrawals from study not clearly explained (1 study);QUADAS limitation: execution of index test and reference standard not described in sufficient detail (1 study) |
